# Ecological not social factors explain brain size in cephalopods

**DOI:** 10.1016/j.isci.2026.116324

**Published:** 2026-07-01

**Authors:** Kiran Basava, Theiss Bendixen, Alexander Leonhard, Nicole Lauren George, Zoé Vanhersecke, Joshua Omotosho, Jennifer Mather, Michael Muthukrishna

**Affiliations:** 1 University of Oxford, Oxford, UK; 2 University of Arizona, Tucson, CA, USA; 3 Aarhus University, Aarhus, Denmark; 4 London School of Economics and Political Science, London, UK; 5 University of Lethbridge, Lethbridge, AB, Canada; 6 New York University, New York, NY, USA

**Keywords:** zoology, evolutionary biology, phylogeny

## Abstract

Social factors have been argued to be the main selection pressure for the evolution of large brains and complex behavior, but many cephalopods live largely solitary, semelparous, short lives. This suggests that the large brains found in cephalopods are not the result of social selection pressures. Here, we derive specific, preregistered predictions from the “Asocial Brain Hypothesis” (ABH; an untested extension of the cultural brain hypothesis formal model), and evaluate those predicted associations using a new comparative dataset on brain size alongside social, ecological, and life history factors. Consistent with the ABH and other hypotheses predicting that ecological factors should be the primary selection pressure with larger brains in more calorie-rich complex ecologies, we find that shallower and benthic (seafloor) habitats are associated with larger brain sizes, and that measures of sociality are not. Our findings are not interpreted as causal, but are consistent with ecological hypotheses for brain evolution.

## Highlights

• Ecological complexity, not social factors, is associated with brain size in cephalopods
• Habitat type is the strongest ecological predictor, rivaling the effect of body size
• Asocial Brain Hypothesis is supported, leading to a general theory beyond social species
• We present a new comparative dataset and phylogeny for coleoid cephalopods

## Graphical abstract

**Figure:**
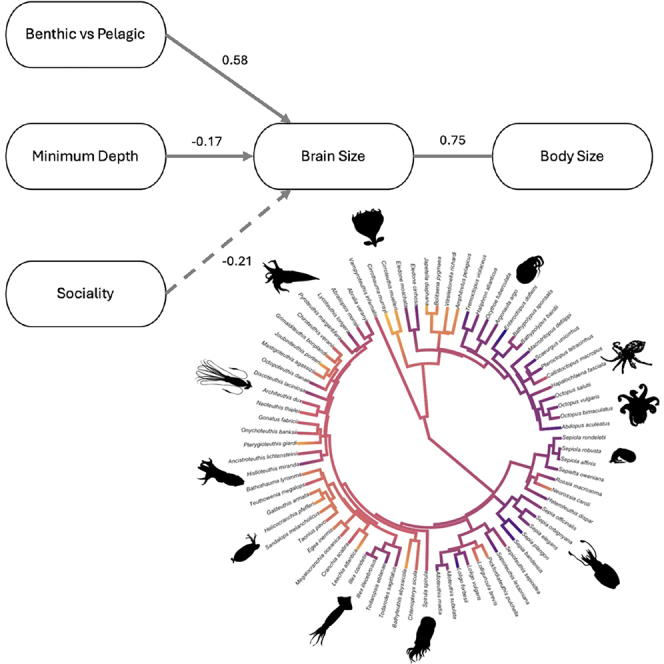

## Introduction

Why do some species evolve much larger brains than others? For decades, researchers have tackled this question by identifying correlations between brain size and various ecological, social, and life-history factors. Competing hypotheses abound – from the social brain hypothesis (SBH) (linking brain size to social group size or other measures of sociality^1^) to ecological-intelligence^2^ and life-history explanations.^3^^,^^4^ But as Shou et al.^5^ note, statistical correlations “on their own do not constitute understanding” (p. 1) We gain understanding only when we uncover mechanistic explanations for those patterns. In other words, correlations suggest possible factors in brain evolution, but by themselves they cannot tell us how or why brains evolved, nor disentangle cause from consequence among multiple confounding variables. This heavy reliance on correlational evidence has left the field with numerous plausible predictors but little in the way of unifying mechanistic theory.

Achieving deeper insight into brain evolution requires the integration of formal theoretical models that go beyond pattern-finding. Mechanistic theory can elevate our inferences by generating explicit predictions to test, thereby moving us from “just-so” correlations to causal hypotheses. Formal mathematical and computational scientific theories provide “a unifying framework”^5^ for disparate findings and can make predictions that are tested and refined through observation and serve as critical “proof-of-concept” tests of verbal hypotheses.^5^ Such formal models act as rigorous examinations of the logical steps in our explanations, much like experiments test empirical hypotheses. Importantly, developing a mathematical model forces clarity about assumptions and causal pathways, often revealing whether a hypothesized mechanism could in principle produce the observed pattern. An additional advantage is that theory can tackle questions where direct experiments are infeasible: “the ability of theory to circumvent practical obstructions of experimental tractability … is a benefit that should not be underestimated”^6^ (p. 5). Even when we cannot manipulate evolutionary pressures in real time, we can use models to explore how evolution might have proceeded under different conditions. In sum, formal theory provides the mechanistic and predictive backbone needed to interpret correlational data in a more meaningful way. That is, we can evaluate the degree to which different models can produce empirically discovered correlations between features of species within a taxon.

Within the study of brain evolution, new theoretical frameworks are emerging to unify the diverse drivers of brain expansion. The motivation for the present study is one such theory developed by Muthukrishna et al.^7^ - the cultural brain hypothesis (CBH). The CBH proposes that brains evolve principally to acquire, store, and manage information. Rather than focusing only on social group size or any single factor, the CBH posits that larger brains are selected for their capacity to handle more information – whether gained through individual (asocial) learning or social learning. This theory captures how cognitive demands (learning skills, innovation, memory) co-evolve with life history and sociality, providing a unifying framework for brain-size variation across taxa. Crucially, the CBH model yields distinct scenarios or “regimes” of brain evolution. The theory was developed to understand the cultural and social pressures typical of humans and primates, hence the term “Cultural Brain”. In the first paper proposing the theory, the CBH predictions were shown to reproduce the correlations between primate brains, group size, innovation, social learning, mating structures, and the length of the juvenile period. The model was subsequently tested with whales and dolphins using a new database created for the purpose of testing the model.^8^ Given the nature of marine research, the data were less rich than with primates, but here, too, the model was consistent with brain size, social structures, and cultural behaviors across cetaceans.

The formal model from which the CBH was derived unexpectedly also predicted a regime with a pathway to big brains driven not by social learning but by asocial information acquisition in complex environments. We call this the “Asocial Brain Hypothesis” (ABH). In this paper, we follow the same protocol used to test the CBH predictions in cetaceans to evaluate if the ABH predictions are consistent with cephalopod data.

The ABH extends the logic of the CBH to species with minimal social learning: it argues that even in largely solitary animals, intense ecological challenges and individual learning opportunities can favor increased brain size as long as learned skills allow an animal to acquire sufficient calories to support the larger brain. In other words, the calorie richness and complexity of the environment, rather than social complexity, becomes the primary selection pressure on brains in asocial lineages. The details of the model are discussed in the next section. In the present study, we examine brain evolution in coleoid cephalopods – a group that offers a compelling asocial contrast to the usual social vertebrate models – to assess whether the ABH’s theoretical predictions are consistent with this lineage.

Octopuses, squids, and cuttlefish have some of the largest brains (relative to body size) among invertebrates and exhibit complex behaviors on par with those of birds and mammals. Most cephalopods present a paradox for social brain theories: they are generally asocial creatures. Many octopus and squid species are short-lived and semelparous (dying after a single reproductive event), largely solitary, and even cannibalistic, which leaves little opportunity for extended parental care, social transmission, social tolerance, intricate dominance hierarchies, or cultural learning. Their large brains clearly did not evolve for managing stable social groups. Why, then, did cephalopods evolve such big brains? The ABH was not developed with cephalopods in mind and therefore does not fully capture some aspects of this group, such as the reproductive cycle. Nonetheless, as Servidio et al.^6^ explain for the role of abstract biological models, it serves as a proof of concept of mechanisms that could generate observed correlations and therefore a guiding framework for data collection and analysis. The ABH makes explicit how if social factors are minimal, then ecological factors, particularly availability of calories with trial and error learned know-how (for how to navigate the complexity to unlock those potential calories) should emerge as the main correlate of brain size.

Motivated by this insight, we compiled comparative data on brain size and a suite of ecological, behavioral, and social variables across 79 cephalopod species. We find that the cephalopod data strongly support this prediction. Species inhabiting calorie-rich complex environments have significantly larger brains, whereas measures of sociality show no such relationship. These results align neatly with the idea that ecological complexity, not social complexity, has been the primary factor associated with cephalopod brain expansion. It is important to note that we interpret these correlations as consistent with a theory that offers a mechanistic explanation, but not as direct causal claims based on the data itself. That is, the positive correlation between benthic living and brain size does not causally demonstrate that a complex habitat selects for bigger brains in real time – many unmeasured factors could be at play, and other models could in theory produce the same predictions. However, such a finding is both motivated by and consistent with the ABH’s mechanistic causal scenario in which calorie richness and ecological challenges create selection pressures favoring cognitive investment. In this way, the correlations serve as empirical support for theoretical predictions in the absence of experimental manipulation, even if there may be other verbal models (see next section and Discussion) and formal theoretical models yet to be developed that show the same pattern of results.

In addition to our overall findings, our study illustrates how formal models and comparative data can work hand-in-hand to advance theories of brain evolution. By illustrating that even an asocial lineage can evolve large brains under the right ecological conditions, we expand the explanatory scope of brain -evolution theory beyond primates and other social animals. More broadly, this work underscores the value of combining mechanistic models with comparative analyses. Such an approach helps unify disparate findings and encourages the development of general frameworks applicable across life’s diversity. In moving beyond simple correlations toward theory-driven analyses, we take a step closer to understanding how and why brains evolve along different paths.

### Prior research and alternative hypotheses

The evolution of large brains across the animal kingdom has been a subject of extensive research, with various social, behavioral, developmental, and ecological factors being proposed as key drivers of this energetically costly adaptation.^9^^,^^10^^,^^11^^,^^12^ Comparative studies have identified a range of predictors for larger brains, including group size, social structure, sexual competition, parental care, length of the juvenile period, predation pressures, diet, locomotion, and sensory capabilities.^13^^,^^14^^,^^15^^,^^16^^,^^17^ Historically, such research has been biased toward groups similar to humans, both taxonomically (e.g., primates and other mammals) and behaviorally, with high levels of sociality (e.g., birds). Therefore, social factors have become the dominant explanatory predictor, after initial work by Jolly^18^ and Humphrey^19^ argued for social complexity as a driver for intelligence among primates, and the hypothesis gained support on the basis of data from mammals and birds.^16^^,^^20^^,^^21^^,^^22^ The SBH in particular was developed to explain the evolution of large brains in primates,^1^ and argues that larger brains evolved to cope with the complex demands of social group living, such as cooperation, deception, and competition,^23^^,^^24^^,^^25^ as well as pair bonding and social cohesion.^21^^,^^26^

The SBH has been tested among various taxa with nuanced results. Some findings have implicated the quality of social relationships rather than group size. Among birds, for example, species with small social groups (5–30 individuals) seem to have larger relative brain sizes than solitary species or those forming large groups.^26^^,^^27^^,^^28^ An analysis of sexual competition and brain size in bats found larger brains among species with monogamous rather than promiscuous females.^29^ A study on African cichlid fishes included sex-specific analyses and found that females providing sole parental care had larger brains than those in species with biparental care, with no effect of care type on male brain size.^30^ A comparative study of cetaceans found a quadratic relationship with group size, where the largest-brained species (and those with larger social repertoires) were associated with mid-sized social groups rather than smaller/loose aggregations or megapods.^8^

In other taxa, no relationship or even a reverse relationship has been found between social measures and brain size. Among carnivorans, an analysis using fossil data indicated that, contrary to a previous analysis of mammalians,^20^ sociality was not linked with increased encephalization across clades and this relationship was likely driven solely by canids.^31^ A study of a rodent group, African mole-rats, showed larger absolute brain size and neuron numbers among solitary species, with the authors highlighting the entanglement of body size with both brain size and the evolution of group living.^32^ Among non-avian reptiles, relative brain size was not correlated with habitat complexity, and in contrast with findings in primates, solitary species had larger brains than social ones.^33^

Most of the research effort has been on vertebrates. Social insects are a notable exception, with some studies exploring the relationship between sociality and brain size. Due to the nature of labor division in many of these species, sociality could be expected to have an inverse effect on brain size; however, differences in expected cognitive load due to variation in how specialization takes place in a colony– e.g., by age where an individual progresses through all tasks in some groups, changes in how labor is divided as a colony grows in size, and variation in colony size over time and within species– complicate this prediction.^34^^,^^35^ This very different expression of sociality, as well as the presence of high intraspecific variation in relative brain volume and the contribution of particular cellular mechanisms to behavioral variation apart from volumetric measures of brain size, may explain the inconsistent results for tests of the SBH among insect groups.^34^^,^^35^

Taken together, these disparate findings highlight how expressions of social behavior with differing cognitive demands - e.g., dominance hierarchies in primates, cooperative breeding in birds, and colonial societies of related individuals in insects - across and within different taxa complicate any clear relationship with behavioral complexity, cognition, and brain size. A recent meta-analysis of the social intelligence hypothesis (here encompassing the Machiavellian intelligence hypothesis and the SBH) found broad support for a “significant positive relationship between sociality and cognition across interspecific, intraspecific and developmental studies”.^36^ The authors note that despite criticisms of measures of brain size and cognitive ability, there was not a significant difference in effect sizes across studies with different measures of cognition, or between studies of primates vs. non-primates. However, they also note that the vast majority of studies have been carried out on primates, other mammals, and birds, and that insects - the only invertebrates in the studies surveyed - were highly underrepresented given the number of taxa. Therefore, it remains unclear the extent to which sociality is linked with cognition outside these heavily studied taxonomic groups.

Two other potential explanatory variables for the evolution of large brains are ecology and life history and development. Research on the importance of ecological complexity in driving brain size has focused on the cognitive abilities required for innovation, problem-solving, and foraging for difficult-to-extract or high-energy foods, and the larger brains that can be supported by ecologies with more calories and energy availability. An early study by Clutton-Brock and Harvey^37^ found differences in brain size (relative to body size) across primates according to ecological differences, including diet (e.g., folivores and frugivores) and home range size. A later study of primates tested measures of ecological vs. social variables,^38^^,^^39^^,^^40^ finding frugivorous diets (requiring spatial memory and extractive foraging and providing higher energy) were a better predictor of larger brains than social group size, while there was no support for the SBH. A comparative study of mammals and birds^38^^,^^39^^,^^40^ found that while experimental measures of self-control were predicted by absolute brain size, they were not strongly linked with residual brain size or at all with social group size, casting doubt both on the link between sociality and cognition and the validity of residual brain size as a measure of cognition. Another study found that brain size among primates was predicted by manipulation complexity, which was also linked with tool use and extractive foraging.^38^^,^^39^^,^^40^

The ‘cognitive buffer hypothesis’ focuses on life history and development, arguing that the behavioral flexibility afforded by large brains will allow organisms to respond to socioecological adversity, with the fitness cost of additional developmental time offset by reduced extrinsic mortality, resulting in overall longer lives and time for reproduction.^41^ There has been some support for this hypothesis, with studies of mammals,^41^^,^^42^^,^^43^ birds,^41^^,^^42^^,^^43^ and amphibians^43^ showing a coevolutionary relationship between large brains and longer lifespans. Kaplan and Robson^4^ use a formal model and empirical data to demonstrate a coevolutionary relationship between brain size and longevity in primates, a correlation also shown in parrots.^44^ However, a recent analysis of cetaceans showed a negative relationship between brain size and lifespan.^45^

This body of research has been largely empirical, yielding disparate and sometimes contradictory relationships between a variety of variables.^13^^,^^20^^,^^42^ One way to unify this disparate body of work is through the use of general theories that formally describe the complex co-evolutionary relationships between all relevant variables.

### An attempt at unifying models of brain evolution

**Figure 1:**
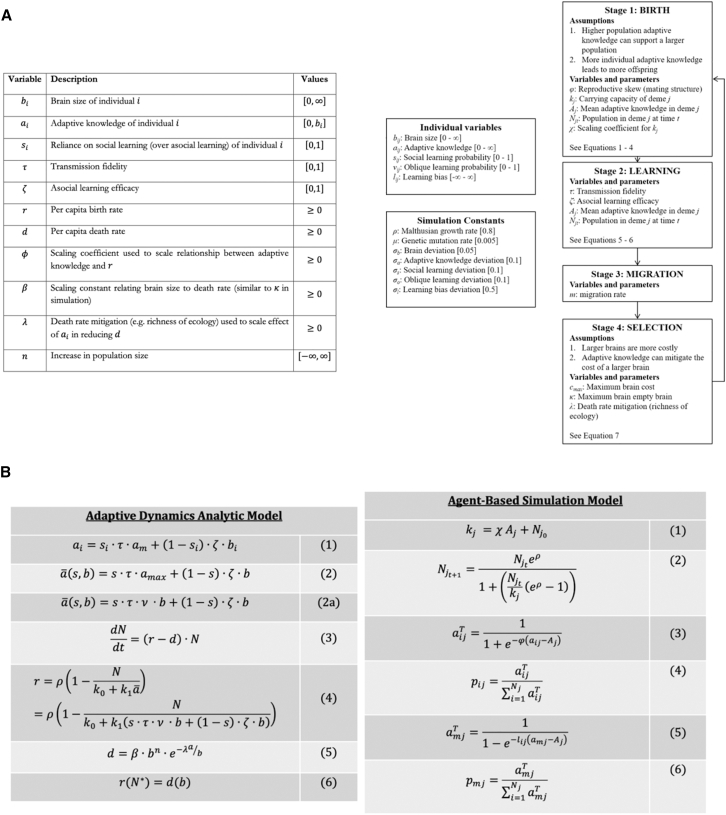
Details of model from which the asocial brain hypothesis, cultural brain hypothesis, and cumulative cultural brain hypothesis are derived (A) Variable definitions for the analytic model, with individuals *i* represented by brain size *b*_i_, adaptive knowledge *a*_i_, and reliance on social over asocial learning *s*_i_ (B) Variable definitions for the agent-based simulation model. The mathematical relationships between variables for the analytic model (left) and agent-based simulation model (right).

**Figure 2:**
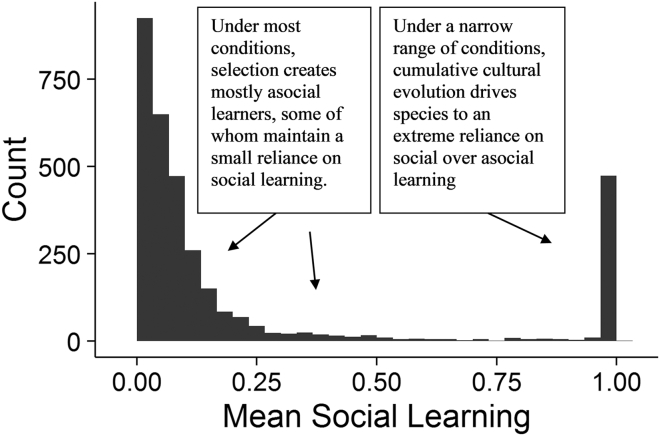
The different equilibrium regimes for reliance on social learning that emerged in the simulation Most conditions select for individuals primarily reliant on asocial learning (left side), while a small range of conditions may create individuals primarily reliant on social learning (right side). A narrow set of conditions leads to an extreme reliance on social learning (far right). These mechanisms identified by the model that lead to these three regimes are the asocial brain hypothesis, cultural brain hypothesis, and cumulative cultural brain hypothesis, respectively.

General theories that formally mathematically or computationally model the causal pathways and coevolutionary relationships can shed light and make clear predictions. One such model was developed by Muthukrishna et al.^7^ The analytic and computational models (see Figures 1 and 2) were developed to incorporate sociality, ecology, and life history into a single framework. It builds on previous work, including the verbal theories of the Cultural Intelligence Hypothesis,^46^^,^^47^ but formalizes assumptions about the relationships between brain size, organismal fitness, and energy requirements. The model revealed two social regimes and an unexpected third asocial regime for the evolution of large brains. The first and second sets of predictions were for social and highly social animals. The “cultural brain hypothesis”, described the social learning path to larger brains, which has been tested with primates^3^ and cetaceans.^8^ The “cumulative cultural brain hypothesis” revealed a narrow set of parameters within the social learning path that would lead to an autocatalytic takeoff consistent with the human pathway characterized by an ever-increasing reliance on culture and technology (see also^48^). The predictions in the third asocial regime, which we label the “ABH”, were untested and therefore motivated the present research on asocial species with a large literature and data collection. Before we present the results, we summarize the assumptions and predictions of the model.

The formal model (from which the Cultural Brain and Asocial Brain Hypotheses are derived) had three key assumptions from which all other relationships emerge.

1. Larger and more complex brains are more costly than less complex brains because they require more calories, take longer to develop, and have organizational challenges. Therefore, increasing brain size/complexity decreases an organism’s fitness; an organism should have the smallest brain possible that allows it to thrive (for simplicity, we assume that brain size, complexity, and organization (e.g., neuronal density) are captured by a single state variable, which we will refer to as “size”). BUT
2. A larger brain correlates with an increased capacity and/or complexity that allows for the storage and management of more adaptive knowledge (adaptive knowledge could potentially relate to locating food, avoiding predators, securing mates, hunting prey, and so on). AND
3. More adaptive knowledge increases an organism’s fitness either by increasing its number of offspring compared to conspecifics and/or by reducing its probability of dying before reproduction. Adaptive knowledge can be acquired socially, through experience, trial and error, and causal reasoning, or socially, by learning from others.

The predictions derived from these assumptions were modeled using two approaches. An analytic approach using an adaptive dynamics evolutionary model^49^ to show how brain size, adaptive knowledge, and reliance on social learning varies as a function of transmission fidelity in social learning (which may be a function of social tolerance, theory of mind, etc.), asocial learning efficacy (which may be a function of learning opportunities, physical ability to manipulate the environment, etc.), and survival returns on adaptive knowledge (via calorie availability as say a function of sunlight and water).

The model leads to 3 key predictions, only the third of which is relevant to the present study on asocial animals.

1. Increased reliance on social learning requires high transmission fidelity (relative to the ability to generate knowledge by oneself).
2. Extreme reliance on social learning requires access to a range of models with different amounts of adaptive knowledge.
3. A greater return on adaptive knowledge (affected by the calorie richness of the environment) increases brain size.

The complexities of co-evolutionary dynamics and more complex social learning strategies were difficult to derive analytically, so instead, a simulation was used to capture these complexities. Figure 1A shows the variables for the analytic and agent-based simulation models, and the stages of the simulation model. Figure 1B shows the mathematical relationships between the variables in the analytic and simulation models. The analytic and simulation solutions can be found in Muthukrishna et al.^7^

The simulation reproduced the results of the analytic model with nuance around social learning strategies. A sweep across the range of parameters suggested that most species have limited social learning (top left of Figure 2), but that within this regime, there was still a pathway to larger brains. This ABH suggests that such “trial and error” learning animals would ignore conspecifics and therefore have a weaker, non-existent, or even negative relationship between brain size and measures of sociality (such as group size or intensity of social interaction) and would have to reduce the length of a juvenile period spent exploring rather than exploiting knowledge. The logic behind this is that trial-and-error learning is less efficient than social learning because the space of possible behaviors and interactions to be learned is much larger than simply learning from a filtered set of socially accumulated actions discovered by other group members; for example, it is faster and easier to be taught something than figure it out on your own. For animals relying on asocial learning, the pressure to begin exploiting learned knowledge is greater, and as a result, we expect that the evolution of larger brains in asocial animals will be primarily constrained by the complexity and available calories in their ecology – there is no corpus of social knowledge that incentivizes a longer learning period.

### Overlap with previous theories

Although the ABH formalizes how asocial learning behavior and life history could interact in certain ecologies to favor the evolution of large brains, it is not the first to suggest this relationship. Previous research has likewise emphasized the role of ecology in behavioral and brain evolution. Among these, Wyles et al.^50^ advanced the “behavioral drive” hypothesis, where animals in a population capable of socially transmitting innovative behaviors can exploit new niches, leading to novel selection pressures that further favor adaptive behaviors and subsequent physiological evolution. However, their focus was on terrestrial vertebrates and discussed social transmission, while the ABH focuses explicitly on asocial animals that learn independently through trial and error. Kaplan and Robson^4^ focus on long-lived, social groups– specifically primates– and find a coevolutionary relationship between brain size and lifespan. They employ a formal model in combination with empirical data and emphasize the importance of ecological conditions in shaping the relative energetic costs of larger brains.

### Cephalopods as a valuable comparative case in brain evolution

With a last common ancestor with humans and other vertebrates over 500 million years ago, cephalopods not only have flexible bodies, but also flexible cognition and behavior. For instance, many species of cephalopods exhibit a variety of foraging and predator-avoidance strategies.^51^ These strategies, such as camouflage, navigation, mimicry, and tool use, can be flexibly adjusted to nearby species or other contextual cues.^52^^,^^53^ Although they do not display the cohesion and complex sociality of many mammalian and bird species, communicative body patterns are used widely across the squid and cuttlefish in mating contexts, where aggressive visual signaling as well as tactical deception have been observed in a range of species.^54^^,^^55^^,^^56^ Many species, especially the well-studied *Octopus vulgaris*, display cognitive abilities such as tool use, navigation, and problem solving, as well as many types of learning.^57^ Playful behavior, an otherwise rare occurrence in the animal kingdom, has also been observed in some octopuses.^58^

Cephalopods, therefore, represent a well-studied, asocial, independent evolutionary origin of intelligence from that of the vertebrate animals we often view as possessing advanced cognition, such as primates, cetaceans, and birds. While gregarious behavior is observed in squid, cephalopods tend to live largely solitary, semelparous, short lives.^51^^,^^52^^,^^57^^,^^59^^,^^60^^,^^61^^,^^62^^,^^63^ Many are cannibalistic, a barrier to social tolerance let alone sociality,^64^ including complex group dynamics or social learning. In our current sample of 79 species where brain data are available, at least incidental cannibalism was observed for 21 species, with data largely unavailable for the remainder. A previous review of a broader set of species found cannibalism in 34 species, associated with various factors including population density, body size, stress, and prey availability.^64^ Cephalopods also exhibit little to no parental care or pair-bonding, often dying after first reproduction.^59^^,^^60^^,^^61^ As these characteristics run counter to established theory and empirical findings from other, more social, vertebrate taxa, cephalopods are a promising animal group on which to evaluate prominent hypotheses on the possible evolutionary drivers of brain size.^62^^,^^63^^,^^65^

As discussed above, explanations focusing on the coevolution of sociality and intelligence do not translate well to cephalopods, particularly the notably solitary octopuses. Researchers have instead focused on explanations involving predation pressures on these soft-bodied organisms and their reliance on extractive foraging in complex oceanic environments.^62^^,^^63^^,^^66^ Packard^67^ extensively documented convergences in cephalopod form and behavior with fish, highlighting the common environment and resource competition that have led to similarities, including in brain development and cognition. Cephalopods would have faced selection pressures to compete for resources, avoid predation by, and prey on fish. Research on cephalopod cognition has illustrated their reliance on exploration and problem-solving, seeking out and integrating information from their environments for extractive foraging, capturing prey, and interacting with conspecifics.^51^ In particular, the extensive repertoire of complex defensive behaviors possessed by extant cephalopods highlights the likely significance of predation in their cognitive evolution.^68^ Phylogenetic studies incorporating data from the fossil record support theories that competition with marine vertebrates during the Mesozoic spurred the evolution of an internal shell and greater locomotion and speed,^69^ which allowed them to colonize a much greater range of ecological niches as well as exposing them to greater predation pressures, increasing selective pressures for sophisticated camouflaging and cognitive flexibility.^62^

Cephalopods also contrast with cetaceans (the marine group previously tested for the CBH^8^), through occupying a far broader range of ocean habitats ranging from shallow coastal water, the open ocean, and the seafloor at a wide range of depths.^70^ Overall, shallow benthic (seafloor) habitats can be considered more calorie-rich, energy available, and biodiverse environments than pelagic (open water) habitats. However, these measures depend on factors such as geographic scale, latitude, depth, substrate, and specific organismal lifestyles (e.g., daily vertical migration from the depths to the surface).^71^^,^^72^ Additionally, much remains to be discovered about organisms inhabiting the deep sea, open ocean, and other environments.^73^^,^^74^ To capture the variety of ecological selection pressures these habitats impose, we include several environmental variables, including benthic vs. pelagic habitats, depth in meters, and latitude (described further in results and methods).

To evaluate the consistency between ABH’s predictions and cephalopod data, we systematically surveyed the cephalopod literature to compile a wide variety of qualitative and quantitative data on extant coleoid cephalopod species’ brain and body size, physiology, ecology, life-history, sociality, and behavior. We read all 3933 papers in the Zoological Record and extracted all available data on 115 variables (see Supplemental Material for full list) for all 79 species for which comparable adult brain size data are available from the year 1866 to July 2020. We also added papers published up to 2024 that contained new brain data. This data revealed large gaps in what we do and do not know about extant coleoid cephalopods. Here we report the results of the 3 variables for which we had full data: ecology (as measured by benthic vs. pelagic habitat), depth (minimum and maximum, with minimum more reliable as maximum depth is more difficult to confirm), and sociality (as measured by categorizing species as solitary or gregarious, with an additional category of tolerant/aggregative in sensitivity checks). The availability of these data allowed us to compare ecology to sociality. The results of other analyses, including those relevant to the theoretical predictions, are less reliable. All other variables with sample sizes are reported in the Supplementary Information (SI).

## Results

The ABH predictions as they apply to cephalopods were pre-registered (https://osf.io/nbwsx). Here we present the key results with the most complete data, comparing ecology against sociality, with the full set of results in the SI. All analyses are Bayesian multilevel models with total central nervous system (CNS) volume as our measure for brain size as the outcome variable. Missing covariates were imputed during model fitting, meaning that available information was used to estimate unobserved values. Where multiple data points were available for the same species, we ran it with the maximum brain size value in the main analysis and with all brain size values in sensitivity checks (reported in the SI) - these did not affect the results. We also ran other robustness and sensitivity checks, including considering the maximum, mean, and minimum of some variables such as depth and age at sexual maturity to assess consistency of observed effects across models (SI). To account for the scaling of brain with body size, each model also includes as a predictor dorsal mantle length (ML) as a standard measure of cephalopod body size.^75^ All continuous variables are logged and standardized (mean 0, standard deviation of (1) and all models incorporate phylogenetic relationships as a correlation matrix to account for evolutionary relatedness between species; for further details on model specification and variable operationalization, see the Methods section. All analysis code and data are available in the supplementary. All analysis code is also openly available at https://github.com/kcbasava/ceph-brain-evolution, and data are also available at cephdata.com and will be posted on Figshare.

**Figure 3:**
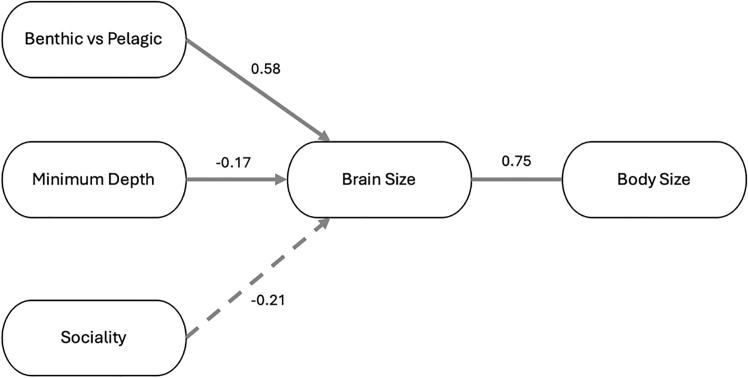
Strongest predictors of brain size found in our analysis The dashed line from sociality indicates that the credibility interval included 0. Note that these are the main effects with phylogenetic and research effort controls.

For each model, we report the point estimate for the mean of the posterior distribution and the lower and upper bounds of the 95% credibility interval (CI). The posterior distribution contains the probability of parameter values conditional on the model and data. The result plots report marginal posterior predictions with covariates held at their mean. A headline summary of the results for the main variables is presented in Figure 3 and Table 1. As ML is reliably large and positively associated with brain size (a posterior mean point estimate of ∼0.75 across models on a logged and standardized scale) with a known biological cause, for ease of interpretation, we interpret the coefficients of our predictors benchmarked relative to this estimate as this is plausibly the largest effect size we could observe (i.e., body size should be most strongly associated with brain size).

**Table 1:** Summary of the results for our main predictor variables, indicating the strength of the relationship relative to that of body to brain size, the direction of the relationship and consistency/lack thereof with the ABH, and our subjective assessment of the reliability of this relationship

Variable	Strength of relationship relative to ML:brain relationship	Sign of relationship	Results consistent with ABH	Strength of evidence^a^	Number of species with data on focal predictor
Benthic (vs. pelagic) habitat	∼⅔	positive	yes	strong	79
Depth	¼ (minimum depth) 18 (maximum depth)	negative	yes	medium	79
Sociality	∼ $\frac{1}{3}$	negative	yes	medium	79

Remaining results using variables with more missing data are reported in the SI.

a Informal strength of evidence assessment based on the amount of missing data, source data quality, effect size, CI ranges, and accuracy of variable definitions, as discussed in the main text.

The relationship between brain size and habitat was two-thirds as large as the relationship between brain size and body size; the relationship with minimum depth was a quarter, maximum depth was an eighth, and the relationship with sociality was a third, but in the negative direction.

### Ecological complexity

For asocial learning species such as cephalopods, the ABH predicts a positive relationship between brain size and richer ecologies across all regimes.^7^ This prediction emerges in the model because richer ecologies with more calories can support larger brains for both social and asocial learners. Additionally, this relationship is expected to be reinforced by a greater behavioral repertoire leading to more access to calories, consistent with ecological explanations for brain evolution.

In our statistical models, ecological complexity was operationalized through: habitat type (benthic, pelagic); minimum and maximum depth in meters and broad categories (shallow, deep, ontogenetic or daily migration); latitude; dietary breadth, measured through the number of taxonomic categories consumed (e.g., crustaceans, gastropods); and number of predators, also measured through the number of taxonomic categories (e.g., other cephalopods, bony fishes).

#### Habitat type

**Figure 4:**
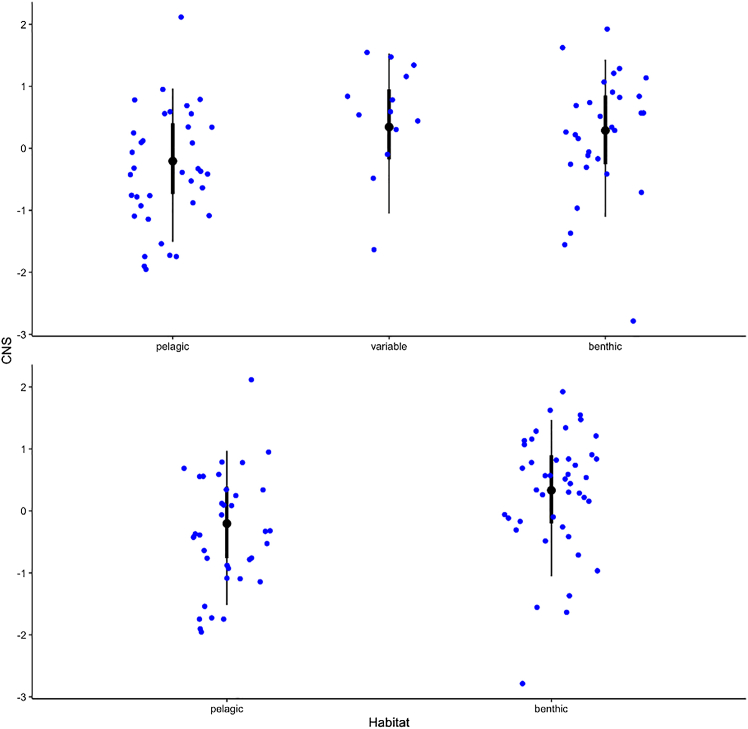
Plot of results from analyses predicting brain size from habitat coded into three or two categorical types Bars show predicted CNS (medians with the highest density continuous intervals) and blue dots observed data for three-category habitat (top) and binary habitat categories (bottom).

We ran an analysis considering habitat as a binary variable (benthic vs. pelagic). The effect of benthic habitats on CNS was positive (0.58 [0.08, 1.07]). To test the robustness of this effect, as lifestyles are not always easily divisible into benthic and pelagic, we also considered a three-category measure of habitat (pelagic, benthic, and variable/near bottom/demersal, which under the binary measure is classified as benthic). The results still revealed a positive effect for near bottom/demersal habitat species (0.64 [0.09, 1.20]) and for non-bottom benthic species (0.54 [-0.02, 1.09]). The estimates for pelagic were somewhat negative in both analyses (-0.42/-0.41 [-1.60/-1.56, 0.79/0.77]). We plot these relationships in Figure 4.

While correlational, the results of the analyses conducted with the first version of habitat codes are consistent with the predictions of the ABH with selection for larger brains in more calorie-rich, complex habitats. However, this is complicated by some species displaying elements of a pelagic lifestyle in addition to interacting with the benthos. Depth range as a potential proxy for ecological complexity was not positively associated with larger brains in our analyses (it did reveal a negative effect, but this was likely driven by higher maximum depth in species with the largest depth ranges). The distribution of depth ranges in meters for each species in the dataset is shown in Figure S2, colored by habitat and ordered in ascending order of relative brain size (CNS/ML). In particular, the plot illustrates how species with relatively larger brains tend to live in benthic or variable habitats.

The phylogenetic analysis of Lindgren et al.^70^ indicated convergent morphological changes in cephalopods with shifts to different oceanic habitats. These included the presence of photophores for bioluminescence in pelagic cephalopods, accessory nidamental glands to protect eggs for species that attach them to substrate, and corneas among benthic taxa for protection from sediment. It is logical that selective pressures of different habitats would likewise influence brain evolution across cephalopod taxa.

#### Depth

Depth measures in our dataset are somewhat imprecise, given that many species have wide and/or variable ranges or engage in vertical migration, and due to the potential biases in measurement methods (e.g., different capture methods in fisheries studies). However, we attempted a rigorous analysis using both minimum and maximum recorded depths, as well as a categorical measure that took into account life history differences in depths occupied. Noting that minimum measures are more reliable as it is easier to measure how shallow a species occurs than how deep, minimum recorded depth had a negative effect (-0.17 [-0.28, -0.04]). Maximum recorded depth also had a negative effect (-0.10 [-0.26, 0.04]), though the CI included 0.

**Figure 5:**
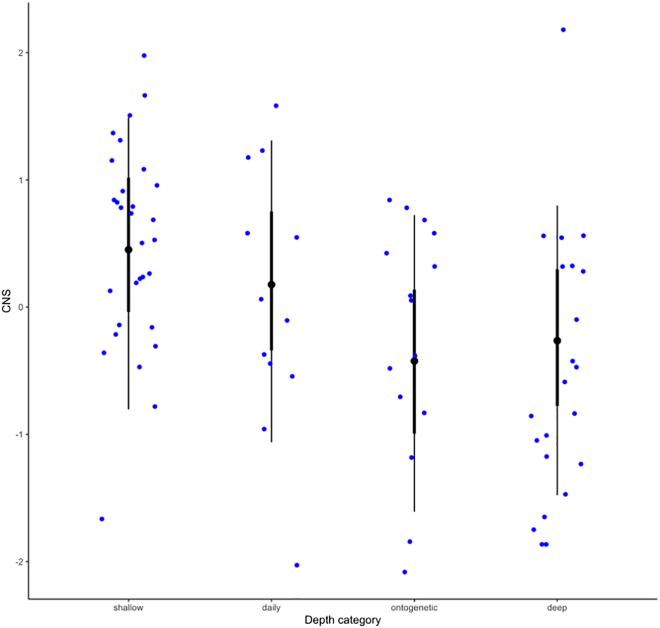
Plot of results from analyses predicting brain size from depth measured in four categories Bars show predicted CNS (medians with the highest density continuous intervals) for different depth categories and blue points show observed data. From left to right: shallow water, daily migration, ontogenetic migration, deep water. CNS is logged and standardized.

Considering depth as a 4-factor categorical variable, categorizing species into shallow water, daily vertical migration, ontogenetic migration (usually descending as they grow from paralarvae/juveniles to adults), and deep-sea also indicated lower CNS for ontogenetically migrating species (-0.85 [-1.35, -0.24]) and deep-sea species (-0.70 [-1.21, -0.20]). The estimate for shallow-water species was centered around 0 (0.15 [-1.00, 1.31]) and for daily vertical migration slightly negative (-0.26 [-1.82, 0.82]). These estimates are plotted in Figure 5.

In summary, the effect of depth is negative, consistent with our predictions, assuming that shallow habitats tend to be more complex than deep-sea ones due to the presence of sunlight and greater energy availability.

### Sociality

**Figure 6:**
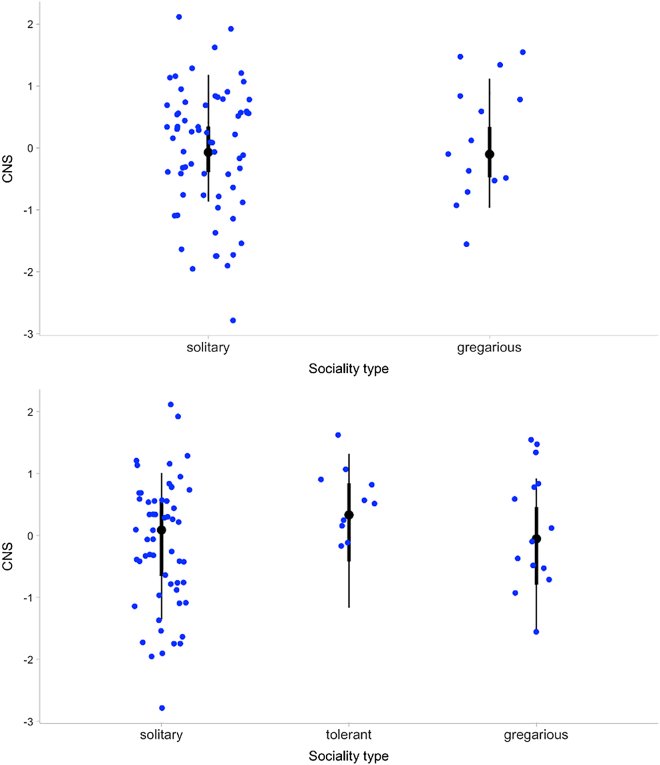
Plot of results from analyses predicts brain size from sociality measured as a binary (top) and 3 categories (bottom) Bars show predicted CNS (medians with the highest density continuous intervals) for different sociality categories and blue points represent observed data. CNS is logged and standardized.

The ABH predicts a weak, non-existing, or even negative relationship between brain size and sociality for cephalopods, as asocial learners do not benefit from a larger group in the way social learners do (larger groups have more models to learn from). This prediction is one of the key differences between the SBH and ABH. Consistent with this prediction, whether species were solitary or gregarious was not positively associated with brain size (Figure S3). Sociality instead had a somewhat negative effect (-0.26 [-0.60, 0.17]), although the CI crosses 0, within the range of the ABH predictions. We also ran an analysis using three categories for sociality rather than the binary measure, with an additional ‘in-between’ code meant to capture species that appear tolerant of or would associate with other individuals close by (coded as solitary in the binary variable). These include some species that have been observed aggregating in the wild or displaying pair-bonding behaviors, such as *Abdopus* species^76^^,^^77^ or that appear unusually tolerant of proximity in lab conditions, such as *Rossia macrosoma*^78^ and *Sepia bandensis*.^79^ The results were broadly similar for social species strictly coded (-0.21 [-0.58, 0.21]). Tolerant species had a slightly higher estimate for brain size (0.37 [-0.02, 0.79]), but interpretation of this result is complicated by the small number of species in this category (11 total) and the heterogeneity of characteristics that could classify species as falling between solitary and gregarious. These results are shown in Figure 6.

A supplementary analysis was also run to look at sociality only among the 54 decapodiforme species (squid, bobtail squid, and cuttlefish) in our dataset. The effect remained negative (-0.24 [-0.76, 0.25]), indicating that this effect was not driven only by larger brains among octopuses, which are predominantly solitary. A corresponding analysis among octopodiformes was not possible, as none were coded as gregarious.

Our sociality findings contrast with many of the predictions and findings from mammals and other social vertebrates. Sociality in cephalopods is strongly phylogenetically conserved, with schooling present exclusively (though not universally) among the decapodiformes. Solitary and social species can generally be clearly distinguished; however, there are many aspects of sociality, such as group sizes and schooling dynamics in the latter, that have not been adequately observed or described in the literature. Cannibalism, common among at least some cephalopods, is another factor that may have obstructed the evolution of richer social lifestyles through inhibiting the possibility of any social trust between conspecifics.^64^ Additional field research is needed to draw more fine-grained conclusions about how sociality interacts with cognition and brain size in cephalopods.

## Discussion

We present the largest and most comprehensive database of cephalopod species where brain data are available. Analyses of this dataset reveal phylogenetically controlled relationships that are consistent with ecological theories of brain evolution, but not consistent with social theories.^1^^,^^21^ Measures of ecological complexity, but not solitary vs. gregarious lifestyles, predicted larger brain sizes in our dataset. This was the case both across species in all three major groups and within squids and cuttlefish, which do display some levels of social grouping and interspecific interactions. Habitat in particular emerged as the variable most reliably associated with brain size in cephalopods, with shallower-living and benthic species tending to have larger brains, since shallower and benthic habitats are more calorie-rich and biodiverse relative to deep-sea and pelagic habitats. For instance, pelagic food webs tend to have lower species diversity than do benthic ones, possibly leading to greater specialization among benthic foragers.^73^^,^^80^ This finding is consistent with the idea that environmental variability selects for greater cognitive capacities and larger brains.^81^ Although we did not evaluate different lobe sizes (which is the subject of a follow up paper), our findings are also consistent with Ponte et al.‘s^82^ assessment of cerebrotypes in relation to phylogeny, development, and environmental niches which emphasized the importance of habitat and life strategies in shaping brain variation across species. Overall, these findings are consistent with hypotheses that have emphasized how large brains may coevolve with the ability for innovative and complex behaviors, allowing animals to survive in variable environments and cope with ecological challenges.^70^^,^^81^

### Limitations and future directions

The main limitation of this analysis is the sparsity of cephalopod data available in the literature, including for species for which brain data are available. Our dataset includes comparative quantitative brain data for 79 species. These are all the species for which brain data are available, but amounts to roughly 10% of the total number (around 820) of extant cephalopod species. To maximize the use of the data we do have, we used a Bayesian multilevel modeling approach so that no data were discarded. Nevertheless, the amount of data does affect the size of the CI, which both emphasizes the strength of the ecological findings, but urges caution for the more limited data availability driving the analyses in the SI.

For example, our measure of predator breadth, which is an important aspect of ecology, suffers from missing data, overgeneralization of taxonomic categories, and difficulty with accurate measurements for many species. More data would be required to create a higher resolution measure of calories and complexity, incorporating temperature, latitude, and oxygen levels affecting diversification, competition, and predator-prey interactions^71^^,^^83^ (see SI for further discussion). However, the categorical measure of deep-sea lifestyles and ontogenetic migration to the depths both showed lower estimates of brain size. Overall, further research on specific environmental aspects of different depths, as well as how variables like predation pressures and prey availability differ for cephalopod species inhabiting benthic or pelagic habitats^68^ will likely shed more light on ecological selection pressures on brain size.

This contrasts with the complete data in habitat type, which is far easier to measure and classify. A far greater amount of lab and field data would be needed to properly test the predictive value of variables like behavioral innovation rate, which has been shown to be associated with brain size in birds and primates.^14^^,^^84^ While our results similarly support some aspects of the cognitive buffer hypothesis,^85^ i.e., selective pressures for cognitive ability from a variable environment, this predicts that large brains will preferentially be selected in longer-lived species,^41^ which does not apply well to short-lived coleoid cephalopods. Further research will help clarify the aspects of these different environments that serve as selection pressures, and how these interact with variables such as predation pressure and diet that were less conclusive in our analyses.

Another possible limitation is that by constraining our analyses to species with presently available quantitative brain data, we risk a selection bias if these particular ‘brain data’ species are exceptional in relevant characteristics compared to non-sampled species. However, the present dataset spans a wide variety of species that are diverse in size and habitat and covers a wide range of families across the major lineages (squid, octopuses, and cuttlefish) of the coleoid cephalopod phylogeny. It therefore seems unlikely that brain size directly influences the probability of sampling, which would be the worst-case scenario from a selection bias perspective.

A further limitation of the current study was the single measurement of brain and body size for the majority of species, which means that intra-species variability was not taken into account, and idiosyncrasies of the individuals that happened to be measured might have affected the validity of our results. To mitigate this, we ran sensitivity checks by including the additional brain and mantle estimates for the 36 species in our dataset with additional (one or two) brain and ML measures. The results of these analyses are summarized in the SI and are consistent with the estimates from our main analyses. There is also the possibility that as methods used to capture brain size differ between older and newer analyses, leading to inconsistencies between the data we combined. As with ecological and behavioral data, future research producing additional measurements for brain size would be valuable to further evaluate processes of brain evolution.

As our analyses used whole brain size as the focal outcome, they also did not take into account measures of brain structure, organization, and neuron numbers, which have been suggested to relate to variation in behavioral repertoires and complexity in cephalopods and other species.^86^^,^^87^ Among cephalopods, this includes research on relative lobe size and how these link with ecological factors.^60^^,^^88^^,^^89^ Ideally, additional work measuring these factors among different cephalopod species will make an analysis incorporating these other potentially important measures possible in the future. More generally, a model-to-data fitting (simulation-based inference) is a valuable next step once richer data coverage exists.

**Figure 7:**
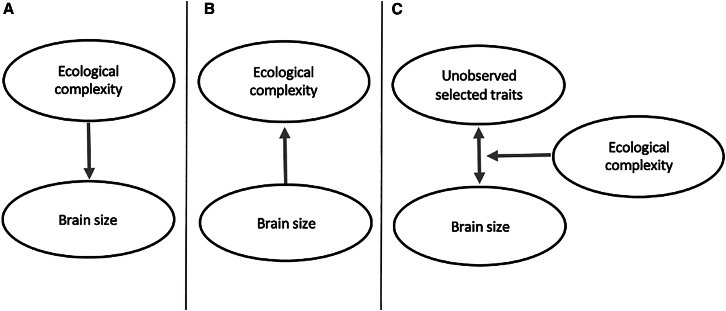
Illustration of alternative hypotheses for results showing a link between brain size and ecology From left to right: (A) ecological complexity selects for larger brains, (B) larger brains enable survival in more complex, energy-rich habitats, or c. ecological complexity is associated with both larger brains and other traits under selection.

Finally, our analysis cannot distinguish among several alternative explanations for the observed link between ecology and brain size. One possibility is that ecological complexity directly selects for larger brains (ecology → brain), as formally modeled in the ABH, and verbally hypothesized by others. However, there are alternative explanations that could also be modeled in future theoretical work. Larger brains, for example, might enable cephalopods to exploit more complex, resource-rich niches (brain → ecology). Or ecological factors could correlate with some other trait that co-evolves with brain size (ecology → (brain ↔ trait)), creating a spurious association. Because our analysis is comparative and correlational, it cannot adjudicate between these scenarios, which we illustrate in Figure 7. The range of possible imagined evolutionary pathways that may lead to big brained cephalopods is also why formal modeling is essential, as it is more generally for theory development in the study of complex systems.^90^ Since all we have are data from extant species, models such as the ABH, that explicitly lay out the outcomes of specific assumptions and mechanisms, are an essential tool to helping us make sense of the variety of empirically discovered relationships reported in the literature.

In summary, we present a novel large comparative dataset of ecological, behavioral, and physiological traits from an exhaustive survey of the literature on cephalopods that can be expanded upon and used for future comparative research. Our analyses of this data provide new evidence for the importance of ecology, rather than sociality, for brain evolution in cephalopods. These findings contrast with the relationship between sociality and brain size among primates and other vertebrate species, highlighting a previously neglected group in this literature and illustrating the potential significance of ecological complexity as a selective pressure. We hope that this dataset and approach motivate more formal theories that capture the evolutionary history and phenotypic constraints specific to cephalopod physiology and life history. Such efforts are the necessary building blocks toward a fully general theory and theoretical framework of brain evolution.^91^

### Limitations of the study

Brain data were available for only 79 of approximately 820 extant cephalopod species, though these span a wide range of sizes, habitats, and phylogeny. Moreover, our Bayesian multilevel modeling approach maximized the use of available data. Most species had only a single brain and body size measurement, precluding analysis of intraspecific variability, though sensitivity checks using additional measurements for 36 species produced consistent results. Our focus on whole brain size did not capture variation in brain structure, lobe organization, or neuron numbers, which may relate to behavioral complexity in cephalopods. Finally, although our results are consistent with the ABH mechanistic model that motivated this empirical effort, the empirical data cannot distinguish whether ecological complexity directly selects for larger brains, larger brains enable exploitation of more complex niches, or ecological factors correlate with other co-evolving traits.

## Resource availability

### Lead contact

Further information and requests for resources should be directed to and will be fulfilled by the Lead Contact, Michael Muthukrishna (michael@muthukrishna.com).

### Materials availability

This study did not generate new unique materials.

### Data and code availability

• All data have been deposited on GitHub and are publicly available as of the date of publication at www.github.com/kcbasava/ceph-brain-evolution.
• All original code has been deposited on GitHub and is publicly available as of the date of publication at www.github.com/kcbasava/ceph-brain-evolution.
• Any additional information required to reanalyze the data reported in this paper is available from the lead contact upon request.

## Acknowledgments

We thank the 10.13039/501100011730Templeton World Charity Foundation for supporting this work (TWCF0464). We would also like to thank Ryutaro Uchiyama for early feedback on the study design and Jan Strugnell for assistance and advice in compiling and constructing the phylogeny.

## Author contributions

K.B.: methodology, software, validation, formal analysis, investigation, data curation, writing – original draft, writing – review & editing, visualization, and project administration, T.B.: conceptualization, methodology, software, validation, formal analysis, investigation, data curation, writing – original draft, writing – review & editing, visualization, supervision, and project administration, A.L.: conceptualization, methodology, validation, investigation, data curation, writing – review & editing, visualization, and project administration; N.G.: investigation and data curation; Z.V.: investigation and data curation; J.O.: investigation and data curation; J.M.: conceptualization, methodology, validation, writing – review and editing, and supervision; M.M.: conceptualization, methodology, validation, software, resources, writing – original draft, writing – review and editing, supervision, project administration, and funding acquisition.

## Declaration of interests

The authors declare no competing interests.

## STAR★Methods

### Key resources table

**Table:** 

REAGENT or RESOURCE	SOURCE	IDENTIFIER
**Deposited data**		

Raw and analyzed data	This paper	Data S1
GenBank	NIH	https://www.ncbi.nlm.nih.gov/genbank/

**Software and algorithms**		

R ×64 4.4.2	R Software Foundation	https://www.r-project.org/
Brms: Bayesian regression models using STAN	Brms package	https://cran.r-project.org/web/packages/brms/index.html
Phytools: Phylogenetic Tools for Comparative Biology (and Other Things)	Phytools package	https://cran.r-project.org/web/packages/phytools/index.html
BEAST2: Bayesian evolutionary analysis by sampling trees	BEAST 2	https://www.beast2.org/
Code and data to replicate analyses	Github	https://github.com/kcbasava/ceph-brain-evolution

### Experimental model and study participant details

Prior to the development of the cephalopod database we present here, the most comprehensive comparative study on cephalopod brain size to date was published as part of a 2007 PhD dissertation by Borelli^92^ and in a subsequent journal article by Ponte and colleagues.^82^ This ambitious and useful contribution had limitations with regard to broader research efforts on comparative brain evolution.

1. The dataset expresses brain size in relative terms (specifically, the size of each brain lobe is computed as a proportion to the whole central nervous system) and does not include absolute brain and body size measurements, which makes these data unsuitable for comparative regression analyses.
2. The dataset does not include social, behavioral, or life-history variables necessary for directly evaluating predictions of the ABH.
3. Due to the nature of available cephalopod phylogenies, Ponte et al.^82^ were forced to discard data on more than half of their 78 species when running phylogenetically-controlled analyses.
4. Upon reviewing Borelli’s primary brain data sources^60^^,^^88^^,^^89^ and their subsequent use in the literature, we found inconsistencies that ultimately required us to conduct a careful, systematic re-evaluation of all data (refer to Supplementary Information for details).
5. The dataset contains brain measures from the juvenile specimens for a few species. As cephalopod brains are still maturing during the juvenile period^93^^,^^94^ and possibly during adulthood,^95^ it is not appropriate to use these for comparative analyses with measures from adult specimens.
6. Considerable advances in empirical work on cephalopods, including additional brain data measurements, have been made since Borelli’s pioneering 2007 PhD data collection.

The present study overcomes these limitations. The dataset we present in this paper was developed with three main goals^66^: (1) to assess key hypotheses on the evolutionary drivers of brain size in cephalopods, starting with the Asocial Brain Hypothesis (ABH); (2) more generally, to move toward a theoretical reconciliation of empirical findings from comparative brain size studies in other animal groups; (3) provide a dataset to the cephalopod research community for further analyses and theory-building. Drawing on the predictions of the ABH as well as previous brain evolution literature, here we empirically assess whether measures of ecology, age at sexual maturity (i.e., length of juvenile period), sociality, and behavioral complexity are associated with brain size in coleoid cephalopods.

We present a new comparative dataset for cephalopods. Brain and body size data were taken mainly from Wirz^89^ and Maddock and Young,^88^ updated by Nixon & Young,^60^ combined with a few newer species-specific brain data estimates,^96^^,^^97^^,^^98^ including Chung et al.^96^^,^^97^ and Montague et al.^98^ We used brain size measurements from adult specimens of identifiable species (79 total). Data on behavior, ecology, physiology, and other traits were collated from a systematic review of articles written on each species on Web of Science, taking into account alternate species names. The bulk of the data was captured up until November 2020, which was followed up by a more targeted data collection effort for particular species and variables up to 2024. We also captured and coded data from major books on cephalopod ecology, physiology, and behavior.^59^^,^^60^^,^^99^^,^^100^^,^^101^^,^^102^

Due to a lack of data on pre-adult stages, we coded our variables focusing on adult brain and behavior. Also due to limitations of the data, we did not distinguish between observations of male or female individuals except in cases of sex-specific variables (e.g., male courtship displays).

We also present a new phylogeny for cephalopods. Although there are available phylogenies of coleoid cephalopod species (e.g.,^70^^,^^103^), the usual approach to comparative analyses would require matching the species in our dataset to those present on a chosen phylogeny and discarding those not included, which would significantly reduce our sample size. As we did not wish to discard any species with brain data, we decided to create an updated phylogeny for our analyses instead. With assistance from Jan Strugnell, we developed a new phylogeny for all 79 species by combining previously published phylogenetic analyses and additional sequence data on GenBank (described in Methods and in Basava et al.^104^).

**Figure:**
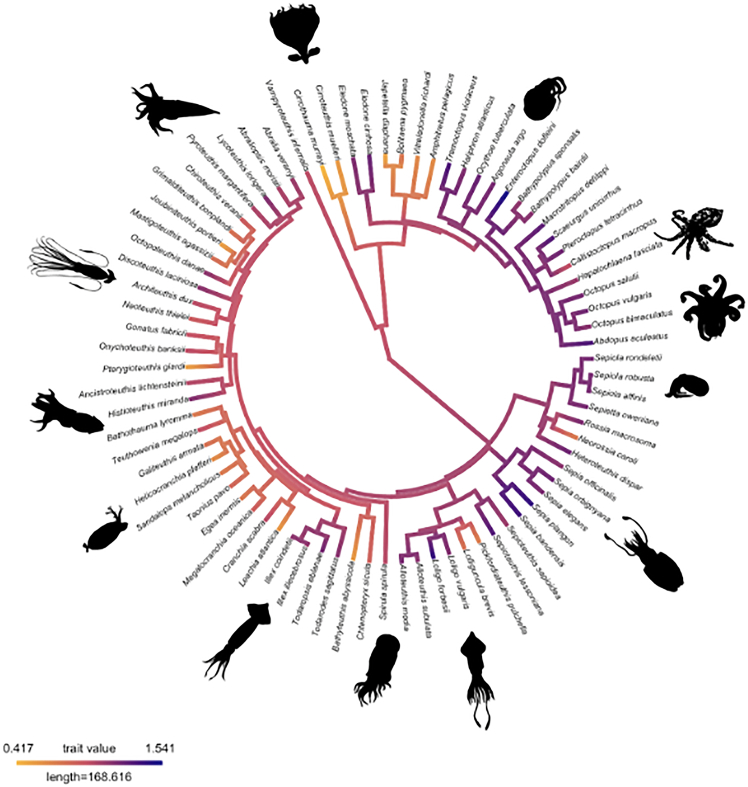
Ancestral state reconstruction of relative brain size (CNS/ML) on a log scale Images from phylopic.org (Keesey 2024).

### Method details

Brain size was operationalized as the total central nervous system (CNS) volume including optic lobes (in millimeters^3^) from the sources listed above. Body size was operationalized as the mantle length from the same specimens in millimeters. In the analyses reported here, we use the maximum recorded total CNS volume and, when available, its associated mantle length size. As sensitivity checks, we re-ran main models with available supplementary CNS data (see https://github.com/kcbasava/ceph-brain-evolution/tree/main/sensitivity-checks).

We operationalized the length of the juvenile period/age of sexual maturity through the oldest age at which a species is not observed to be sexually mature measured in number of days, with lifespan (also in days) included in the models for which this was a predictor. We analyze both the maximum and minimum recorded ages. Sociality was measured as a categorical variable with species coded as solitary (avoiding other conspecifics except for mating), tolerant/aggregative (an in-between category with individuals sometimes observed as congregating outside of reproductive contexts and appearing tolerant of nearby individuals), or gregarious (usual or obligate schooling). This was coded as a binary variable in the main analyses with ‘tolerant’ species coded as solitary/0. Sensitivity checks were run with the three-category measure.

Species were characterized as having benthic or pelagic habitats, with benthic defined as animals living on and/or closely interacting with the seafloor and pelagic animals primarily living in the water column. After further review of these variables, it was decided to create a third category for species observed interacting both with the seafloor and swimming in the water column (demersal/in-between) that would otherwise be classified as benthic. Depth was measured through the minimum and maximum depths in meters at which adult individuals of each species had been recorded, as well as a 4-category variable which classified species as living in shallow waters, undergoing daily vertical migration, migrating to the depth as adults (i.e., ontogenetic migration), and living in the deep sea.

Behavioral complexity was measured through (a) combined social behaviors and cognitive measures, (b) number of defense/antipredator behaviors, and (c) number of foraging behaviors, each used as predictor variables (full description in SI). This measure was intended to capture behavioral complexity both as measured through lab studies of abilities such as learning, memory, and problem solving, as well as through field observations of interactions with conspecifics in the wild, e.g., with deception in mating contexts and communication abilities, for species which have not been studied in one or the other context. Unfortunately, this variable was too sparse to yield results we felt were robust, hence they are not included in the main text and we do not draw theoretical conclusions from them. As we did preregister and run analyses with behavioral complexity as a predictor, for transparency they are included in the SI.

Researcher effort was accounted for by including variables for (1) number of articles published on Web of Science for the species name (or alternative names) and (2) articles read during coding of the dataset. The former was used as the standard variable for research effort while the latter was used for variables more likely to be biased due to frequency of observation (i.e., count variables including dietary breadth, number of predators, or foraging strategies).

The phylogeny was constructed through manually combining topologies from previous phylogenetic studies assembled with assistance from Dr. Jan Strugnell, a specialist in cephalopod genetics, and the branch lengths inferred through a BEAST 2.7.4 analysis via the BEAUti GUI (Bouckaert et al. 2019) with gene sequences from GenBank (Benson et al. 2013). Accession numbers for sequences used and citations for the topology are in Supplementary Data 3 in Data S1. A consensus tree (maximum clade credibility tree, highest product of posterior node probabilities) was obtained using the software TreeAnnotator v2.7.4. Additionally, the posterior distribution of trees from two independent BEAST runs were combined using LogCombiner (Bouckaert et al. 2019) and the combined files were thinned to produce a sample of 100 trees. The list of correlations matrices from this sample of trees were used in supplementary replications of the main analyses to capture uncertainty in branch lengths and polytomy resolutions (Results in Table S3).

### Quantification and statistical analysis

All analyses are Bayesian phylogenetically controlled, multilevel linear regression models and were run using the *brms* package in *R*,^105^ an interface to the probabilistic programming language *Stan*.^106^ They generally follow the operationalization of Bayesian multilevel regression models from McElreath^107^ as implemented in brms by Kurz.^108^ The multilevel structure of the model accounts for the non-independence of the data from species in our dataset through incorporating information on phylogenetic relationships. All models included brain size (as total CNS volume) modeled as a function of body size (as mantle length). Other variables were included as derived from adjustment sets from a directed acyclic graph (DAG) depending on the focal predictor. DAGS describe a hypothesized causal model through nodes (variables) and directed edges (relationships).^109^ The code describing the DAG, including all variables and hypothesized causal paths, are available in the *R* scripts at https://github.com/kcbasava/ceph-brain-evolution. All variables except for habitat, categorical measure of depth, and sociality were standardized (mean centered with a standard deviation of (1) and log-transformed prior to analyses. Priors were chosen to be weakly regularizing to help prevent the model from overfitting to the data.^107^ A consensus phylogeny created from the results of the phylogenetic software (discussed in detail in the Supplementary Information) was incorporated as a covariance matrix following the implementation in Kurz.^110^ Models were fitted according to the following structure:

brm(bf(CNS ∼ mi(ML) + mi(X1) + X2 + effort + (1 | gr(species, cov = cormat))) +

bf(X1 | mi() ∼1 + mi(ML) + X2 + (1 | gr(species, cov = cormat))) +

bf(ML | mi() ∼1 + X2 + (1 | gr(species, cov = cormat))),;

data = d, data2=list(cormat=cormat),

prior = c(prior(normal (0,1), class = Intercept),

prior(normal(0, 0.5), class = b)))

where CNS is total brain volume, ML is mantle length, effort is research effort, X_1_ is a predictor or predictors with missing data defined by the second bf() formula and X_2_ is a predictor or predictors without missing data. ‘Cormat’ represents the correlation matrix for the consensus phylogeny. Models were run for 8000 iterations. As a robustness check accounting for uncertainty in branch lengths, models were also run over a set of 100 phylogenies sampled from the posterior distribution of the phylogenetic inference analysis (described in detail in Supplementary Information) and the results combined using the combine_models() function in *brms*.

Sensitivity checks were run using additional measures of brain data and mantle length for species with measures from multiple individuals to ensure broadly similar results were attained. Sensitivity checks were also run with focal predictors with missing data in the main analyses (age at sexual maturity, predator breadth, diet breadth, hunting repertoire, and defense repertoire) with the missing cases dropped instead of imputed. These analyses did not contradict any of the findings from the imputed analyses (see analyses and supplementary data available at https://github.com/kcbasava/ceph-brain-evolution/tree/main/sensitivity-checks).

#### Data coding

##### Brain data

The brain data for each species are listed in the supplement.xlsx file. We used data from Chung et al. (2020), Chung et al. (2022), Chung et al. (2023), Nixon & Young (2003), Maddock and Young (1987), Montague et al. (2022), and Wirz (1959). We cite Maddock and Young (1987) as the original source and the edited version from Nixon and Young (2003) when applicable. We excluded Frösch (1971) because they strictly studied hatchlings. When there were brain data available from multiple specimens, we used measures from the specimen with the largest estimate for our analysis as this was most likely to be an adult. For our outcome variable, we use the total central nervous system volume including the optic lobe. The inclusion of optic lobes in CNS volume is indicated in the column name in Data S2.

Note that in their Table 2.3, Nixon and Young (2003) seemed to have mixed up the sources – the italicized data are from Wirz (1959), not Frösch (1971).

Wirz (1959): The CNS data (Table II, p. 90) from Wirz are volumes (in mm3) without the optic lobes for the biggest and smallest animals measured for each species, respectively. To get total CNS, we take the CNS reported from Table II and multiply it with the percentages of the optic lobe (OPL) from Table V: Total CNS = CNS + (OPL∗CNS)/100.

• Brain data for Brachioteuthis riisei, Chtenopteryx sicula and Octopoteuthis sicula from Wirz (1959) were excluded as they were from juveniles.

Montague et al. (2023): Reports volume for a long list of lobes.

• We’re interested in total CNS size (“SUM”) which is reported in the supplement material page e2. It is an average of the eight specimens used in the article. Mantle length of the eight specimens were not measured but estimated to be around 6 cm (*pers. comm.*), which is consistent with adult mantle length estimates from other sources (60–90 mm; Thompson, 2017).

Chung et al. (2020): Reports lobe sizes and total CNS volume as well as mantle lengths.

Chung et al. (2022): Reports both total CNS size incl. optic lobes and CNS size excl. optic lobes.

Chung et al. (2023): Reports both total CNS size incl. optic lobes and CNS size excl. optic lobes as well optic lobes and vertical lobe sizes.

• We used total CNS sizes including optic lobes from Chung et al. 2022 and 2023, and total CNS volume and mantle lengths from Chung et al. 2020.

#### Miscalculation in chung et al. (2023)

As noted above, the size figures reported for each lobe in Tables III and V in Wirz (1959) are not absolute volumes but instead percentages of the whole brain (excluding the optic lobe). However, in their Table S2, Chung et al. (2023) take these percentages as *volumes*. To verify, consider O. vulgaris. Wirz (1959) reports in Table IV the volume of the whole brain excluding the optic lobe (column 1; see also Table II), the percentage of the optic lobe to the whole brain (column 2; see also Table III), and the absolute volume of the optic lobe (column 3). To get total CNS in absolute sizes from Wirz (1959) and accounting for the fact that Wirz report optic lobe size for only a single lobe in Table III, we need the following formula:

TotalCNS=CNS+(OPL∗CNS)/100

where CNS is the whole brain size excluding the optic lobe reported in Table II and OPL is the optic lobe percentage reported in Table V (or, alternatively, the single optic lobe percentage reported in Table III multiplied by 2). For the largest specimen of O. vulgaris (Table I, column 1), this means: 242.6 + (122∗242.6)/100 = 538.572 mm3. If we subtract from that the whole brain size excluding optic lobe and divide by two (two optic lobes), we get the absolute volume reported for a single optic lobe for O. vulgaris in Table IV: (538.572–242.6)/2 = 148 mm3.

This miscalculation accounts for the discrepancies between our brain size estimates and those cited from Wirz (1959) in Chung et al. (2023). For instance, for S. elegans, Chung et al. (2023, Table S2) reports a total CNS (“CNS”) of 493 mm3, whereas our calculation (for the largest specimen) yields a more modest estimate of 32.5 + (296∗32.5)/100 = 128.7 mm3.

##### Deviations from the preregistration

Species exclusions: We originally included brain data for *Idiosepius paradoxus* from Koizumi et al. (2016), which although reported as a juvenile fell within the ML ranges at maturity from our literature review. However, we later realized that they had excluded the optic lobe and other parts of the brain from their measure of brain size and it was therefore not appropriate to include in our dataset. We also realized that the measure of brain size for *Octopus cyanea* was reported by the source (Chung et al. 2022) as being from a juvenile specimen. After the exclusion of *Octopus cyanea* and *Idiosepius paradoxus* the number of species in our dataset was 79 (down from the 81 reported in our preregistration).

Measurements for statistical models: Behavioral complexity was originally going to be operationalized through a polychoric principal component of summed behavioral categories: number of foraging/hunting strategies, number of antipredator strategies, summed social behaviors including communication behaviors and mating strategies, and summed cognitive repertoire measured through lab studies on learning and other abilities. However, these variables did not load well onto a single category after imputing missing data (<0.5 proportion variance explained). Instead, number of foraging behaviors and number of defense/antipredator behaviors were each considered as individual predictors. We also attempted to combine social behaviors and cognitive measures into a single variable as a measure of cognition, but ultimately decided the data were too unbalanced in the quality of records and nature of research effort (i.e., lab vs. field) across species, measured too many different behaviors and capacities, and were overall too sparse to serve as an effective measure to capture true interspecific differences in cognition. The better recorded variables for learning and cognition were only available for 7–8 species (e.g., long-term memory and classical conditioning) and for 10–15 species for social behaviors recorded in the wild (e.g., 12 species for positive male behaviors toward females; 9 species for agonistic communication in non-reproductive contexts), and it is not clear whether these capacities and behaviors across lab and field studies can be conceptualized as a single measure.

##### Variable definitions

Total CNS volume_1 (Total central nervous system volume (mm3) -- largest reported): Our outcome variable of brain size, measured in millimeters.^3^ CNS.1 in st.cephdat.

Mantle length_1 (brain size specific): Our measure of body size; dorsal mantle length measured in millimeters. ML.1 in st.cephdat.

Depth: Maximum or minimum depth at which the species has been caught or observed in meters. depth.max, depth.min, depth.mean in st.cephdat.

Depth range: difference between maximum and minimum depths. depth.range in st.cephdat.

Habitat: whether the species is primarily pelagic (living in the open water column) or benthic (mainly living on and interacting the the seafloor). A third category was created for demersal/intermediate habitat organisms that displayed elements of both pelagic and benthic lifestyles. benthic and habitat3 in st.cephdat.

Latitude: Minimum, maximum, mean, and range (difference between maximum and minimum) in degrees recorded for species. As continuous variables were logged prior to standardization for analyses and latitude contains negative values, 90 was added to all minimum and maximum latitude variables so that 0 would be furthest south and 180 would be furthest north. pos.latmax, pos.latmean, pos.latmin in st.cephdat.

Age of sexual maturity: Maximum and minimum age at which the species has been recorded reaching the age of sexual maturity in days (conversion from months to days will be made by multiplying the number of months with 30.4375, which is the average number of days per month including leap year). matage.max and matage.min in st.cephdat.

Lifespan: Life span max (days) is the maximum recorded life-span. Senescence is included in this number. All measurements in time are in days (Conversion from months to days will be made by multiplying the number of months with 30.4375. lifespan.max and lifespan.min in st.cephdat.

Dietary breadth: Originally coded continuously from 1 upwards. ‘Fish’ otherwise unspecified, or an order or family with unspecified genus, were counted as 1. This was then collapsed into a categorical variable from 1 to 10, summed of the following prey categories: Gastropods, Bivalves, Chitons, Cephalopods, Decapod crustaceans, Other crustaceans, Bony fish, Other fishes, Other invertebrates, Other vertebrates. diet.breadth in st.cephdat.

Number of predator groups: Coded 1–9 summing presence of the following categories: Decapod crustaceans, other crustaceans, cephalopods, other molluscs, bony fishes, other fishes, mammals, birds, other (not fish, mammal, bird) vertebrates. predator.breadth in st.cephdat.

Sociality type: solitary (1)- individuals are generally alone in the wild apart from mating; associative/tolerant (2)- individuals may sometimes be found together, appear tolerant of closeby conspecifics (somewhat of an ‘everything else' category between solitary and social); gregarious (3)- aggregations in large units and/or obligatory schoolers. sociality3 in st.cephdat.

Sociality type (binary): solitary (categories 1 and 2 above); gregarious (category 3 above). sociality.bin in st.cephdat.

Research effort (Web of Science): Citations, species name and alternative name in topic 1866–2020). We captured the research effort from the 15th to the 22nd of July 2020 b y searching for the species name and alternative spellings in the Zoological Records. The number refers to the number of hits. Wos in st.cephdat.

Research effort (Articles read): Articles available for access and reviewed for each species (this includes articles not found relevant and not cited in the dataset). articles.read in st.cephdat.

Defense repertoire: Combined total of antipredator behavior types (e.g., camouflage, burying in sand, inking, etc.) and strategies (behaviors altered according to predator or context). For species without recorded use of ink as a defense but known to possess an ink sac, 1 was added to this repertoire *unless* the species would not have data otherwise, in which case imputation was used (in an effort to mitigate bias against less-observed species). defense.repertoire in st.cephdat.

Foraging repertoire: Combined total of hunting and feeding behaviors including types of behavior, prey manipulation, signaling, and hunting and feeding strategies (behaviors altered based on prey species or context). foraging.repertoire in st.cephdat.

Depth categories: 1 = shallow-water species, 2 = diel vertical migration, 3 = ontogenetic migration, 4 = deep-water species. depth_cat in st.cephdat.

#### Causal graph

To correctly include covariates in our analyses, we created a directed acyclic graph (DAG) of all the variables with CNS as the outcome variable and focal predictors as the exposure. The code for the DAG and the adjustment sets (also in the dataprep.R script in the GitHub repository) is below and visualized in Figure S1.

#DAG code----

cephdag <- dagitty(“dag {

ML - > CNS

behavior -> CNS

benthic -> CNS

depth -> CNS

diet -> CNS

latitude -> CNS

lifespan -> CNS

maturity -> CNS

phylogeny -> CNS

predators -> CNS

sociality -> CNS

latitude -> ML

phylogeny -> ML

depth -> ML

latitude -> behavior

WoS -> behavior

phylogeny -> behavior

depth -> behavior

phylogeny -> lifespan

predators -> lifespan

WoS -> lifespan

ML - > lifespan

benthic -> lifespan

depth -> maturity

benthic -> maturity

WoS -> maturity

latitude -> maturity

lifespan -> maturity

phylogeny -> maturity

ML - > maturity

WoS -> diet

ML - > diet

benthic -> diet

depth -> diet

latitude -> diet

phylogeny -> diet

ML - > predators

WoS -> predators

depth -> predators

latitude -> predators

benthic -> predators

benthic -> sociality

depth -> sociality

WoS -> sociality

phylogeny -> sociality

phylogeny -> latitude

WoS -> latitude

benthic -> latitude

WoS -> depth

benthic -> depth

phylogeny -> depth

phylogeny -> benthic

}”)

adjustmentSets(cephdag, outcome=“CNS”, exposure=“depth”).

#{ WoS, benthic, phylogeny }.

adjustmentSets(cephdag, outcome=“CNS”, exposure=“benthic”).

#{phylogeny}.

adjustmentSets(cephdag, outcome=“CNS”, exposure=“latitude”).

#{ WoS, benthic, phylogeny }

adjustmentSets(cephdag, outcome=“CNS”, exposure=“diet”).

#{ ML, WoS, benthic, depth, latitude, phylogeny }

adjustmentSets(cephdag, outcome=“CNS”, exposure=“sociality”).

#{ WoS, benthic, depth, phylogeny }

adjustmentSets(cephdag, outcome=“CNS”, exposure=“maturity”).

#{ ML, WoS, benthic, depth, latitude, lifespan, phylogeny }

adjustmentSets(cephdag, outcome=“CNS”, exposure=“behavior”) #encompasses cognition, defense, and foraging

#{ WoS, depth, latitude, phylogeny }

#### Additional analyses

Overall, whether a species occupied a primarily benthic or pelagic habitat was a reliable predictor of brain size with a posterior mean estimate of approximately 0.5 for benthic habitats in focal models, although with relatively wide CIs. For comparison, the point estimate for mantle length - a strong predictor of brain size - is approximately 0.75 across models (Table 1). The habitat variable is complete in our dataset, and it is generally clear from the scientific literature whether species primarily live in the benthos or open water column, although there is ambiguity with a few species that may primarily live and/or hunt in different habitats and have not been sufficiently observed (e.g., the bobtail squid *Heteroteuthis dispar* and some loliginid squids like *Loligo vulgaris* and *Loligo forbesii*). That said, the effects in our 3-category habitat model taking into account these edge cases and the consistent positive effect of benthic habitat across models indicate that benthic and pelagic lifestyles were a likely selection pressure for brain size in cephalopods.

##### Latitude

Latitude was measured through minimum and maximum recorded ranges of species in degrees. As this variable contains negative values, to calculate mean latitude, 90 was added to all the minimum and maximum latitude variables so that 0 would be furthest south and 180 would be furthest north prior to logging and standardizing it as with the other continuous variables. This was used as an ecological control variable in most of the models testing other predictors.

To specifically test the effects of distance from the equator, the absolute value of the average of the original maximum and minimum latitude measures was used. Distance from the equator did not suggest any effect (-0.03 [-0.16, 0.10]). Latitude range, as one measure of environmental variability, had a slightly negative effect (-0.18 [-0.34, -0.01]).

##### Dietary breadth and predation pressure

Dietary breadth was not associated with a larger brain size (0.02 [-0.20, 0.25]). The interaction with habitat was weakly negative for pelagic (-0.09 [-0.46, 0.31]) and weakly positive for benthic (0.13 [-0.27, 0.52]).

Number of predator taxa was weakly positive (0.08 [-0.11, 0.27]). When considered in interaction with habitat, this effect was close to 0 among pelagic species (0.04 [-0.18, 0.25]) and somewhat positive among benthic species (0.24 [-0.10, 0.54]).

Dietary breadth in isolation was not a clear predictor of brain size with an estimate centered evenly around 0. For the interaction with habitat, the estimate was positive for benthic species with an effect size around ⅕ that of brain-body size (although with relatively wide CIs). Dietary breadth is a variable with inexact measurement methods (sum of taxonomic categories of prey species, with a large amount of variation in each category) and high potential for missing data even with species observed from stomach contents. We also needed to impute values for nearly a third of species. With more systematically collected observational data, the relative effects of both diet breadth and type on brain size could be examined with more precision. The effect of predator breadth was more reliably positive with the bulk of the posterior distribution being positive, which was also stronger for the interaction with habitat; however, similar to dietary breadth, there was a significant amount of missing data. Like diet and number of defensive and foraging behaviors, this is also a variable dependent on observations in the wild or data from stomach content of predator species, so further field research will provide a more accurate assessment of predation pressure across species and how this variation affects brain sizes. See Figure S2.

##### Age at sexual maturity

The ABH predicts a weak or non-existent relationship between brain size and age of sexual maturity relative to lifespan. This prediction emerges for the cephalopods because asocial learners need to move from exploration to exploitation faster, given that asocial learning is less efficient than social learning. In contrast, for social learning species, the juvenile period is extended and age of sexual maturity is delayed to allow for more time to efficiently learn from other members of the group.

In line with this prediction, we found no consistent relationship between brain size and age of sexual maturity (Figure S2). On the one hand, maximum recorded age at sexual maturity had a positive effect (0.33 [0.05, 0.56]). On the other hand, sensitivity checks were run with mean and minimum recorded age of sexual maturity; mean was also positive (0.26 [-0.05, 0.54]) but the minimum was negative (-0.11 [-0.40, 0.19]), and both 95% CI included 0. Data on this variable is sparse and somewhat unreliable; as it mostly comes from indirect measures and different methods may be used to determine whether an individual is mature (e.g., body size, egg development in females), there is inconsistency in how age of sexual maturity is identified across species. Figure S3 shows the both the relationship between sociality and CNS and minimum and maximum age of sexual maturity and CNS.

##### Behavioral repertoire

The ABH predicts a positive relationship between brain size and behavioral repertoire, as for both social and asocial learners larger brains can store and manage more adaptive information leading to a larger set of flexible behaviors.

Overall, our analyses did not support this prediction, as we did not find evidence for a positive relationship between our measures of behavioral complexity and brain size. The estimates for defense repertoire and foraging repertoire were centered around 0 and slightly negative (-0.04 [-0.25, 0.23]) and (-0.06 [-0.30, 0.18]).

Therefore, our proxies for behavioral complexity did not show a consistent effect on brain size. This contradicts both our predictions and previous findings of links between behavioral complexity and brain size in other specie. However, these variables had large amounts of missing data and biases in the distribution of observed behaviors across species.

##### Sensitivity analyses

For 35 of the 79 species, there was more than one brain size estimate. In all cases for the main analyses, we used the largest brain size estimate available. However, here we ran sensitivity analyses to confirm that including these smaller brain size estimates would not change the overall findings. We also ran a sensitivity check with selected models removing S. Lessoniana, which was labeled as a juvenile specimen but whose mantle length falls within the adult size range of that species (per our literature review). We compare these to the main analysis in Table S2, with a full coefficient table of mean estimates and 95% CIs in Table S3.

## Supplemental information

Document S1. Figures S1–S3 and Tables S1–S3

Data S1. All data and code needed to replicate analyses
