# Supplementary material for: Ecological not social factors explain brain size in cephalopods
Source: iScience. 2026 Jul 1;29(7):116324. doi: 10.1016/j.isci.2026.116324 (PMC13343137; doi:10.1016/j.isci.2026.116324)
Supplement: Document S1. Figures S1–S3 and Tables S1–S3 [file mmc1.pdf]

iScience, Volume *29*

Supplemental information

Ecological not social factors

explain brain size in cephalopods

Kiran Basava, Theiss Bendixen, Alexander Leonhard, Nicole Lauren George, Zoé Vanhersecke, Joshua Omotosho, Jennifer Mather, and Michael Muthukrishna

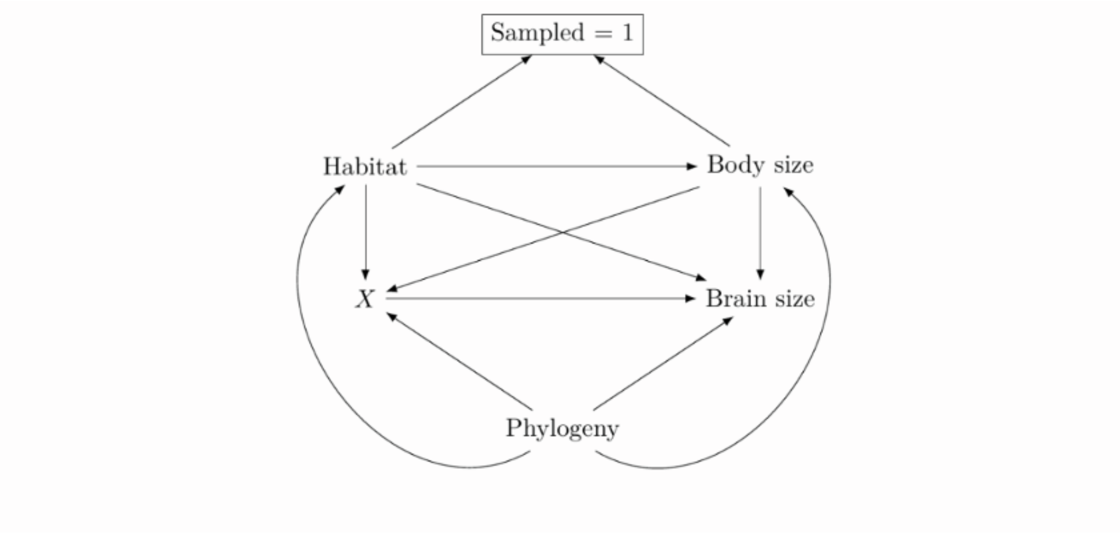

Figure S1. Directed acyclic graph (DAG) of the hypothesized causal structure of the data-generating processes. X refers to our vector of focal predictors. Our estimand is X → Brain size. This DAG is a simplified portrayal of the hypothesized causal pathways for sampling selection bias; for statistical models refer to the adjustment sets derived from the main DAG of variables analyzed (https://github.com/kcbasava/ceph-brain-evolution/blob/main/dataprep_cephs.R).

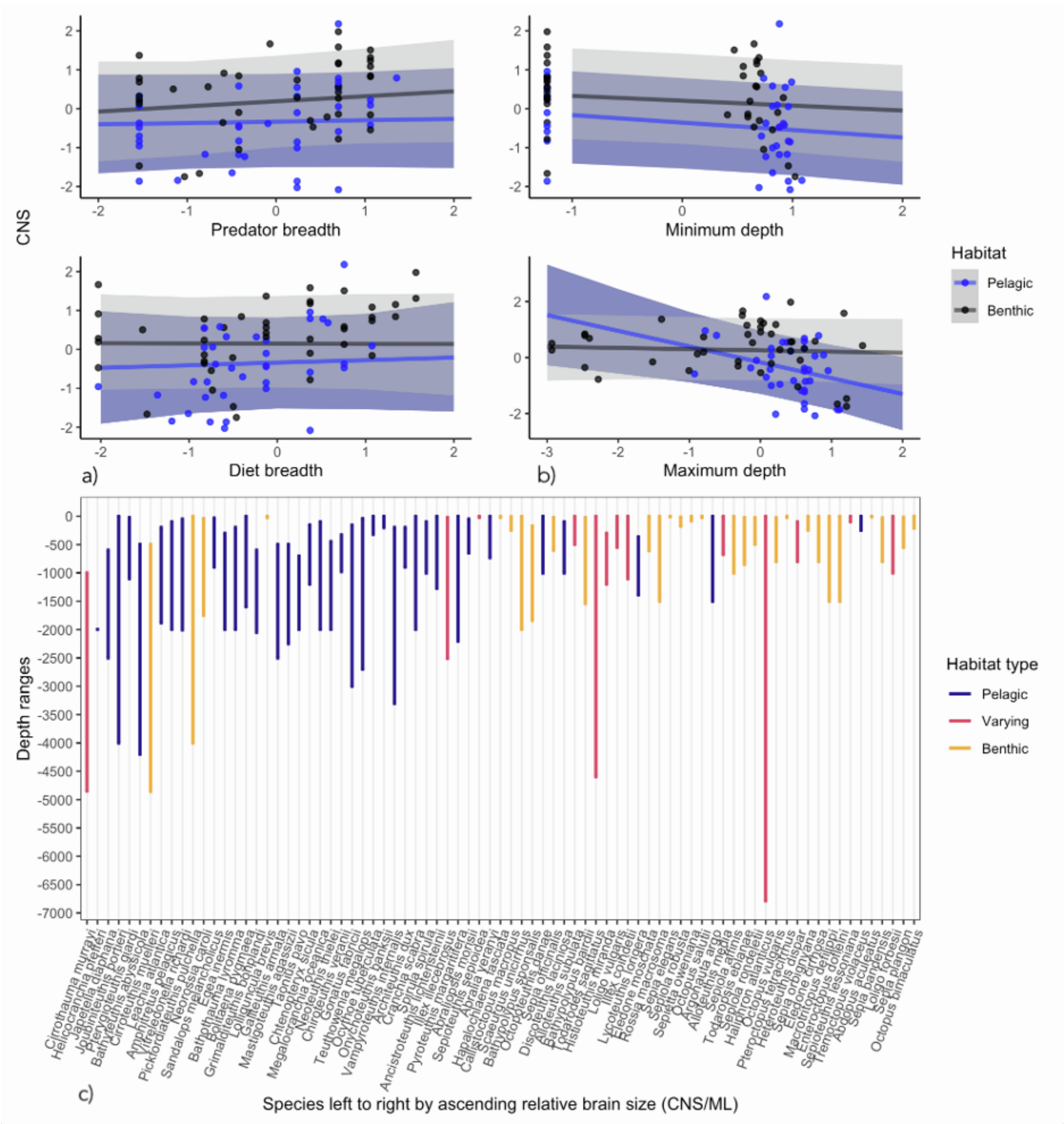

Figure S2. (a) Posterior estimates for CNS predicted by predator breadth (top) and dietary breadth (bottom). CNS and depth, predator breadth, and diet breadth are logged and standardized. (b) Posterior estimates for CNS predicted by minimum (top) and maximum (bottom) recorded depth among pelagic (blue) and benthic (grey) species. Lines indicate posterior medians and shaded areas highest density continuous intervals. CNS and depth are logged and standardized. (c) Depth ranges for species, ordered left to right by ascending brain size (CNS/ML) and colored by habitat type.

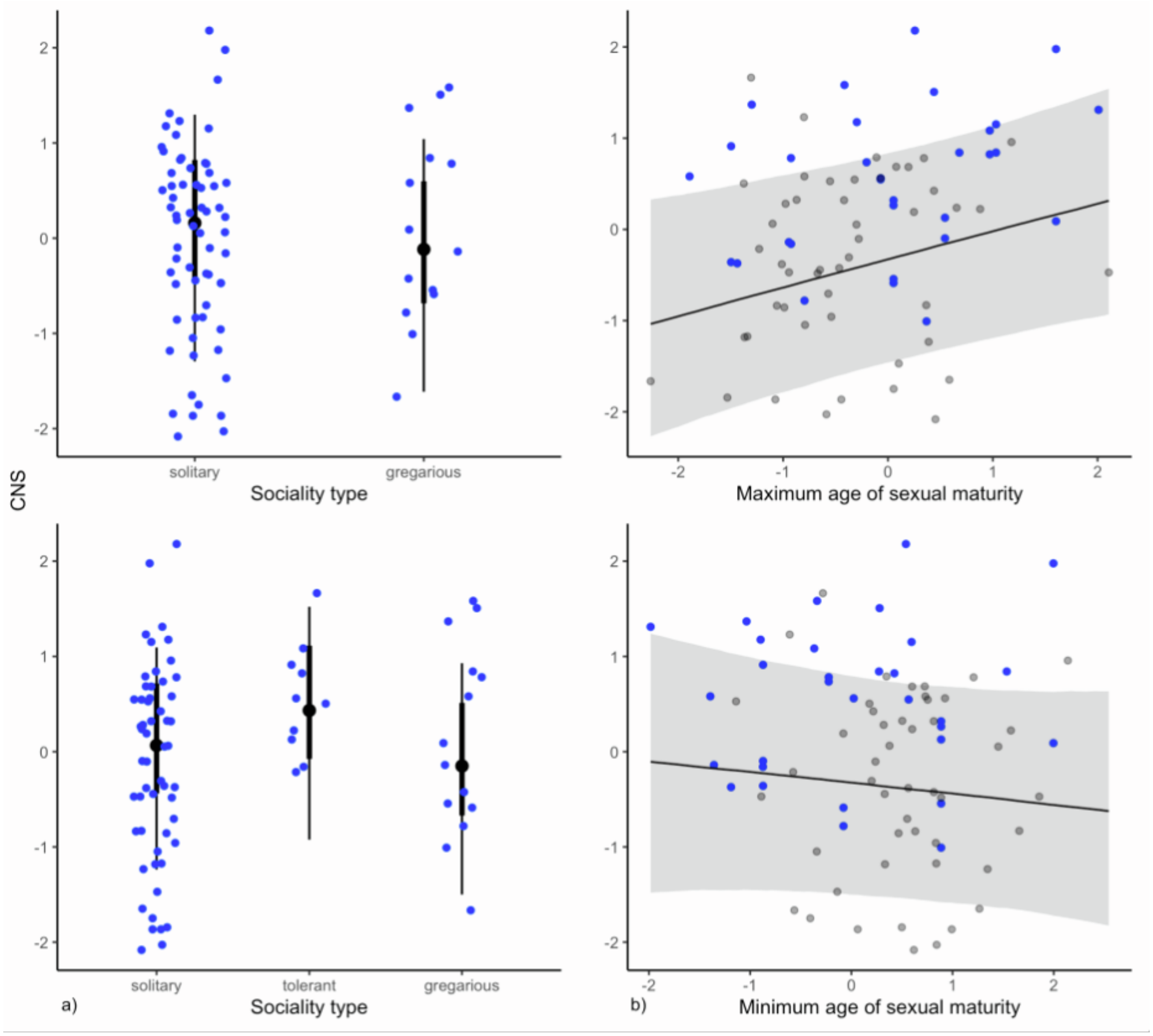

Figure S3. (a) Posterior predicted CNS (medians with highest density continuous interval) for each category of sociality. Top shows results for solitary vs. gregarious categories; bottom is solitary, tolerant/in-between, and gregarious categories. CNS is logged and standardized. (b) Posterior predicted CNS for maximum age of sexual maturity (top) and minimum age (bottom). Black lines indicate posterior medians and gray shaded area highest density continuous intervals. Blue dots are observed data. Grey dots are imputed values (posterior medians) for species missing data. CNS and ages are logged and standardized.

Table S1. Summary of the results for our supplementary predictor variables, indicating the strength of the relationship relative to that of body to brain size, the direction of the relationship and consistency/lack thereof with the ABH, and our subjective assessment of the reliability of this relationship.

*Informal strength of evidence assessment based on amount of missing data, source data quality, effect size, CI ranges, and accuracy of variable definitions, as discussed in the main text. "Minimum", "maximum" and "mean" refers to the minimum, maximum and mean of minimum and maximum recorded values.

Variable	Strength of relationship relative to ML:brain relationship	Sign of relationship	Results consistent with ABH	Strength of evidence*	Number of species with data on focal predictor
**Number of predator groups**	<¼ (conditional on habitat); otherwise 0	positive (conditional on habitat)	yes (conditional on habitat); otherwise no	weak	64
**Age of sexual maturity**	<¼ (minimum); <¼ (mean); ~⅓ (maximum)	negative (minimum); positive (mean and max)	yes	weak	31
**Foraging behaviors repertoire**	**~0**	-	no	weak	34
**Dietary breadth**	~0	-	no	weak	51
**Defensive behaviors repertoire**	~0	-	no	weak	43

Table S2. Sensitivity to different brain size estimates.

Variable	Main Analysis	Sensitivity Analysis (including other brain estimates)	Sensitivity Analysis (removal of S. lessoniana)
Benthic vs Pelagic	0.58 [0.08, 1.07]	0.50 [0.06. 0.94]	0.56 [0.04, 1.07]
Near bottom/demersal<br>Benthic<br>vs Pelagic	0.64 [0.09, 1.20]<br>0.54 [-0.02, 1.09]<br>-0.42 [-1.60, 0.79]	0.57 [0.06, 1.10]<br>0.44 [-0.03, 0.92]<br>-0.51 [-1.49, 0.46]	–
Minimum Depth	-0.17 [-0.28, -0.04]	-0.13 [-0.26, -0.01]	–
Min Depth x Pelagic	-0.19 [-0.37, -0.01]	-0.19 [-0.38, 0.01]	-0.19 [-0.36, -0.03]
Min Depth x Benthic	0.06 [-0.19, 0.33]	0.10 [-0.17, 0.37]	0.04 [-0.18, 0.32]
Maximum Depth	-0.10 [-0.26, 0.04]	-0.24 [-0.38, -0.10]	–
Max Depth x Pelagic	-0.56 [-0.98, -0.14]	-0.55 [-0.90, -0.19]	-0.58 [-0.99, -0.16]
Max Depth x Benthic	0.52 [0.07, 0.97]	0.36 [-0.03, 0.74]	0.54 [0.11, 0.98]
Ontogenetic migrating<br>Deep-sea<br>Shallow-water<br>Daily vertical migration	-0.85 [-1.35, -0.24]<br>-0.70 [-1.21, -0.20]<br>0.15 [-1.00, 1.31]<br>-0.26 [-1.82, 0.82]	-0.76 [-1.33, -0.19]<br>-0.59 [-1.02, -0.16]<br>-0.04 [-0.98, 0.84]<br>-0.22 [-0.73, 0.33]	–
Sociality	-0.26 [-0.60, 0.17]	-0.15 [-0.52, 0.21]	-0.25 [-0.59, 0.14]
Social (strict)	-0.21 [-0.58, 0.21]	-0.12 [-0.48, 0.22]	–
Tolerant	0.37 [-0.02, 0.79]	-0.28 [-0.06, 0.63]	–
Decapodiformes	-0.24 [-0.76, 0.25]	-0.24 [-0.76, 0.25]	–
Distance from Equator	-0.03 [-0.16, 0.10]	0.00 [-0.13, 0.14]	–
Latitude range	-0.18 [-0.34, -0.01]	-0.15 [0.08, -0.30]	–
Dietary Breadth	0.02 [-0.20, 0.25]	0.00 [-0.24, 0.23]	–
Dietary x Pelagic	-0.09 [-0.46, 0.31]	0.01 [-0.11, 0.11]	0.06 [-0.34, 0.45]
Dietary x Benthic	0.13 [-0.27, 0.52]	0.05 [-0.10, 0.26]	-0.06 [-0.44, 0.31]
Number of predators	0.08 [-0.11, 0.27]	0.09 [0.05, 0.14]	–
Predators x Pelagic	0.04 [-0.18, 0.25]	0.05 [-0.07, 0.13]	0.05 [-0.18, 0.28]
Predators x Benthic	0.24 [-0.10, 0.54]	0.11 [-0.02, 0.24]	0.15 [-0.17, 0.47]
Max Sexual Maturity	0.33 [0.05, 0.56]	0.25 [0.18, 0.36]	–
Mean Sexual Maturity	0.26 [-0.05, 0.54]	0.32 [0.22, 0.41]	0.26 [-0.03, 0.50]
Min Sexual Maturity	-0.11 [-0.40, 0.19]	-0.00 [-0.22, 0.14]	–
Defense Repertoire	-0.01 [-0.24, 0.23]	-0.04 [-0.25, 0.23]	
Foraging Repertoire	-0.10 [-0.32, 0.15]	-0.06 [-0.30, 0.18]	

Table S3. Coefficient table of mean estimates and 95% CIs for variables (rows) and corresponding models (columns). It should be noted that the ‘average’ column is mean of posterior means of all models and coefficients should not be interpreted equivalently across models and the meaning will differ depending on included covariates (see code for DAG in Supplementary Information). Remaining coefficients and models are in Supplementary Table 1.

Predator breadth estimate		0.036						0.036
Predator breadth 95% CI		-0.183 to 0.246						
Predator breadth:benthic estimate		0.241						0.241
Predator breadth:benthic 95% CI		-0.102 to 0.537						
Mean latitude estimate	0.076	0.086		0.046	0.075	0.066	0.039	0.065
Mean latitude 95% CI	-0.081 to 0.23	-0.058 to 0.227		-0.102 to 0.192	-0.064 to 0.213	-0.075 to 0.206	-0.101 to 0.172	
Sociality estimate			-0.215					-0.215
Sociality 95% CI			-0.578 to 0.168					

Diet breadth 95% CI					-0.2 to 0.254
Max lifespan estimate		-0.209			
Max lifespan 95% CI		-0.475 to 0.087			
Min lifespan estimate	0.225				
Min lifespan 95% CI	-0.101 to 0.515				
Max age at maturity estimate		0.304			
Max age at maturity 95% CI		0.044 to 0.532			
Min age at maturity estimate	-0.083				
Min age at maturity 95% CI	-0.378 to 0.204				
Predator breadth estimate				0.084	
Predator breadth 95% CI				-0.108 to 0.272	
Mean latitude estimate	0.006	0.039			0.065
Mean latitude 95% CI	-0.165 to 0.173	-0.11 to 0.193			-0.088 to 0.218
Tolerant sociality estimate			0.185		
Tolerant sociality 95% CI			-0.237 to 0.637		
Strict gregarious estimate			-0.189		
Strict gregarious 95% CI			-0.566 to 0.211		

Supplemental Reference List

Bouckaert, R. R., & Drummond, A. J. (2017). bModelTest: Bayesian phylogenetic site model averaging and model comparison. *BMC Evolutionary Biology*, *17*(1), 42. https://doi.org/10.1186/s12862-017-0890-6

Bouckaert, R., Vaughan, T. G., Barido-Sottani, J., Duchêne, S., Fourment, M., Gavryushkina, A., Heled, J., Jones, G., Kühnert, D., Maio, N. D., Matschiner, M., Mendes, F. K., Müller, N. F., Ogilvie, H. A., Plessis, L. du, Popinga, A., Rambaut, A., Rasmussen, D., Siveroni, I., … Drummond, A. J. (2019). BEAST 2.5: An advanced software platform for Bayesian evolutionary analysis. *PLOS Computational Biology*, *15*(4), e1006650. https://doi.org/10.1371/journal.pcbi.1006650

Castiglione, S., Tesone, G., Piccolo, M., Melchionna, M., Mondanaro, A., Serio, C., Di Febbraro, M., & Raia, P. (2018). A new method for testing evolutionary rate variation and shifts in phenotypic evolution. *Methods in Ecology and Evolution*, *9*(4), 974–983. https://doi.org/10.1111/2041-210X.12954

Charif, D., Clerc, O., Frank, C., Lobry, J. R., Necşulea, A., Palmeira, L., Penel, S., & Perrière, G. (2023). *seqinr: Biological Sequences Retrieval and Analysis* (4.2-30). https://cran.r-project.org/web/packages/seqinr/index.html

Chung, W.-S., Kurniawan, N. D., & Marshall, N. J. (2020). Toward an MRI-Based Mesoscale Connectome of the Squid Brain. *IScience*, *23*(1), 100816. https://doi.org/10.1016/j.isci.2019.100816

Chung, W.-S., Kurniawan, N. D., & Marshall, N. J. (2021). Comparative brain structure and visual processing in octopus from different habitats. *Current Biology*, S0960982221015323. https://doi.org/10/gnkdkb

Chung, W.-S., López-Galán, A., Kurniawan, N. D., & Marshall, N. J. (2023). The brain structure and the neural network features of the diurnal cuttlefish Sepia plangon. *IScience*, *26*(1), 105846. https://doi.org/10.1016/j.isci.2022.105846

Clarke, M. R., & Fitch, J. E. (1979). Statoliths of Cenozoic teuthoid cephalopods from North America. *Palaeontology*, *22*(2), 479–511. https://oceanrep.geomar.de/id/eprint/34692/

Douglas, J., Zhang, R., & Bouckaert, R. (2021). Adaptive dating and fast proposals: Revisiting the phylogenetic relaxed clock model. *PLOS Computational Biology*, *17*(2), e1008322. https://doi.org/10.1371/journal.pcbi.1008322

Edgar, R. C. (2004). MUSCLE: multiple sequence alignment with high accuracy and high throughput. *Nucleic Acids Research*, *32*(5), 1792–1797. https://doi.org/10.1093/nar/gkh340

Fuchs, D., Bracchi, G., & Weis, R. (2009). NEW OCTOPODS (CEPHALOPODA: COLEOIDEA) FROM THE LATE CRETACEOUS (UPPER CENOMANIAN) OF HÂKEL AND HÂDJOULA, LEBANON. *Palaeontology*, *52*(1), 65–81. https://doi.org/10.1111/j.1475-4983.2008.00828.x

Fuchs, D., & Weis, R. (2008). Taxonomy, morphology and phylogeny of Lower Jurassic loligosepiid coleoids (Cephalopoda). *Neues Jahrbuch Für Geologie Und Paläontologie - Abhandlungen*, *249*(1), 93–112. https://doi.org/10.1127/0077-7749/2008/0249-0093

Koizumi, M., Shigeno, S., Mizunami, M., & Tanaka, N. K. (2016). Three-dimensional brain atlas of pygmy squid, *Idiosepius paradoxus* , revealing the largest relative vertical lobe system volume among the cephalopods. *Journal of Comparative Neurology*, *524*(10), 2142–2157. https://doi.org/10.1002/cne.23939

Lemoine, F., Correia, D., Lefort, V., Doppelt-Azeroual, O., Mareuil, F., Cohen-Boulakia, S., & Gascuel, O. (2019). NGPhylogeny.fr: new generation phylogenetic services for non-specialists. *Nucleic Acids Research*, *47*(W1), W260–W265. https://doi.org/10.1093/nar/gkz303

Lindgren, A. R., Pankey, M. S., Hochberg, F. G., & Oakley, T. H. (2012). A multi-gene phylogeny of Cephalopoda supports convergent morphological evolution in association with multiple habitat shifts in the marine environment. *BMC Evolutionary Biology*, *12*(1), 129. https://doi.org/10.1186/1471-2148-12-129

López-Córdova, D. A., Avaria-Llautureo, J., Ulloa, P. M., Braid, H. E., Revell, L. J., Fuchs, D., & Ibáñez, C. M. (2022). Mesozoic origin of coleoid cephalopods and their abrupt shifts of diversification patterns. *Molecular Phylogenetics and Evolution*, *166*, 107331. https://doi.org/10.1016/j.ympev.2021.107331

Maddock, L., & Young, J. Z. (1987). Quantitative differences among the brains of cephalopods. *Journal of Zoology*, *212*(4), 739–767. https://doi.org/10.1111/j.1469-7998.1987.tb05967.x

Montague, T. G., Rieth, I. J., Gjerswold-Selleck, S., Garcia-Rosales, D., Aneja, S., Elkis, D., Zhu, N., Kentis, S., Rubino, F. A., Nemes, A., Wang, K., Hammond, L. A., Emiliano, R., Ober, R. A., Guo, J., & Axel, R. (2023). A brain atlas for the camouflaging dwarf cuttlefish, Sepia bandensis. *Current Biology*, *33*(13), 2794-2801.e3. https://doi.org/10.1016/j.cub.2023.06.007

Nixon, M., & Young, J. Z. (2003). *The brains and lives of cephalopods*. Oxford University Press.

Paradis, E., & Schliep, K. (2019). ape 5.0: an environment for modern phylogenetics and evolutionary analyses in R. *Bioinformatics*, *35*(3), 526–528. https://doi.org/10.1093/bioinformatics/bty633

Sanchez, G., Fernández-Álvarez, F. Á., Taite, M., Sugimoto, C., Jolly, J., Simakov, O., Marlétaz, F., Allcock, L., & Rokhsar, D. S. (2021). Phylogenomics illuminates the evolution of bobtail and bottletail squid (order Sepiolida). *Communications Biology*, *4*(1), 1–9. https://doi.org/10.1038/s42003-021-02348-y

Sanchez, G., Setiamarga, D. H. E., Tuanapaya, S., Tongtherm, K., Winkelmann, I. E., Schmidbaur, H., Umino, T., Albertin, C., Allcock, L., Perales-Raya, C., Gleadall, I., Strugnell, J. M., Simakov, O., & Nabhitabhata, J. (2018). Genus-level phylogeny of cephalopods using molecular markers: current status and problematic areas. *PeerJ*, *6*, e4331. https://doi.org/10.7717/peerj.4331

Schweigert, G., & Fuchs, D. (2012). First record of a true coleoid cephalopod from the Germanic Triassic (Ladinian). *Neues Jahrbuch Für Geologie Und Paläontologie - Abhandlungen*, *266*(1), 19–30. https://doi.org/10.1127/0077-7749/2012/0258

Strugnell, J. M., Norman, M. D., Vecchione, M., Guzik, M., & Allcock, A. L. (2014). The ink sac clouds octopod evolutionary history. *Hydrobiologia*, *725*(1), 215–235. https://doi.org/10.1007/s10750-013-1517-6

Thompson, L. E. (2017). *Testing the Optomotor Response in Sepia bandensis* [Honors Thesis]. Georgia Southern University.

Wirz, K. (1959). Etude biometrique du Systeme nerveux des cephalopodes. *Bull Biol*, *93*, 78–117.
